# Neoadjuvant programmed cell death 1 blockade combined with chemotherapy for resectable esophageal squamous cell carcinoma

**DOI:** 10.1136/jitc-2021-003497

**Published:** 2022-01-12

**Authors:** Weixiong Yang, Xiangbin Xing, Sai-Ching Jim Yeung, Siyu Wang, Wenfang Chen, Yong Bao, Fang Wang, Shiting Feng, Fang Peng, Xiaoyan Wang, Shuling Chen, Minghui He, Ning Zhang, Honglei Wang, Bo Zeng, Zhenguo Liu, Biniam Kidane, Christopher W Seder, Kazuo Koyanagi, Yaron Shargall, Honghe Luo, Sui Peng, Chao Cheng

**Affiliations:** 1 Department of Thoracic Surgery, Sun Yat-sen University First Affiliated Hospital, Guangzhou, Guangdong, China; 2 Department of Gastroenterology, Sun Yat-sen University First Affiliated Hospital, Guangzhou, Guangdong, China; 3 Department of Emergency Medicine, The University of Texas MD Anderson Cancer Center, Houston, Texas, USA; 4 Department of Thoracic Surgery, Sun Yat-sen University Cancer Center, Guangzhou, Guangdong, China; 5 State Key Laboratory of Oncology in South China, Collaborative Innovation Center for Cancer Medicine, Guangzhou, Guangdong, China; 6 Department of Pathology, Sun Yat-sen University First Affiliated Hospital, Guangzhou, Guangdong, China; 7 Department of Radiotherapy, Sun Yat-sen University First Affiliated Hospital, Guangzhou, Guangdong, China; 8 Institute of Precision Medicine, Sun Yat-sen University First Affiliated Hospital, Guangzhou, Guangdong, China; 9 Department of Radiology, Sun Yat-sen University First Affiliated Hospital, Guangzhou, Guangdong, China; 10 Department of Nuclear Medicine, Sun Yat-sen University First Affiliated Hospital, Guangzhou, Guangdong, China; 11 Division of Interventional Ultrasound, Sun Yat-sen University First Affiliated Hospital, Guangzhou, Guangdong, China; 12 Department of Liver Surgery, Sun Yat-sen University First Affiliated Hospital, Guangzhou, Guangdong, China; 13 Department of Surgery, Max Rady College of Medicine, University of Manitoba, Winnipeg, Manitoba, Canada; 14 Department of Cardiovascular and Thoracic Surgery, Rush University Medical Center, Chicago, Illinois, USA; 15 Department of Gastroenterological Surgery, Tokai University School of Medicine, Isehara, Japan; 16 Division of Thoracic Surgery, McMaster University/St. Joseph’s Healthcare Hamilton, Hamilton, Ontario, Canada; 17 Clinical Trials Unit and Institute of Precision Medicine, Sun Yat-sen University First Affiliated Hospital, Guangzhou, Guangdong, China

**Keywords:** immunotherapy, clinical trials as topic, drug therapy, combination, tumor microenvironment, therapies, investigational

## Abstract

**Background:**

Programmed cell death 1 (PD-1) blockade induces tumor regression in patients with advanced esophageal squamous cell carcinoma (ESCC); however, little is known about the efficacy of PD-1 blockade as neoadjuvant therapy in resectable ESCC. We aim to assess the safety and feasibility of using the combination of neoadjuvant PD-1 blockade with chemotherapy in patients with ESCC.

**Methods:**

Patients with previously untreated, resectable (stage II or III) ESCC were enrolled. Each patient received two 21-day cycles of neoadjuvant treatment with camrelizumab, nab-paclitaxel, and carboplatin before undergoing surgical resection approximately 6–9 weeks after the first cycle.

**Results:**

Between January 2020 and September 2020, 37 patients were screened, of whom 23 were enrolled. The neoadjuvant therapeutic regimen had an acceptable side effect profile, and no delays in surgery were observed. Severe (grade 3–4) treatment-related adverse events included neutropenia (9 of 23, 39.1%) and leukopenia (2 of 23, 8.7%). The objective response and disease control rates were 90.5% and 100%, respectively. Twenty patients received surgery, and R0 resection was achieved in all cases. Five (25%) patients had a pathological complete response (PCR) and 10 (50%) patients had a major pathological response. The proportion of patients with a high tumor mutation burden and a high expression of programmed death-ligand 1 (PD-L1) in primary tumor was significantly higher in the PCR group than in the non-PCR group (p=0.044). The number of infiltrating PD-L1^+^ CD163^+^ cells was significantly lower in the PCR group than in the non-PCR group after treatment (p=0.017).

**Conclusions:**

Neoadjuvant camrelizumab plus carboplatin and nab-paclitaxel had manageable treatment-related adverse effects and induced an objective response in 90.5% of patients, demonstrating its antitumor efficacy in resectable ESCC.

**Trial registration number:**

ChiCTR2000028900.

## Introduction

Esophageal cancer (EC) is the sixth leading cause of cancer-related mortality in the world.^1^ Esophageal squamous cell carcinoma (ESCC) is the predominant subtype of EC in the Asian populations.^2^ China has a high prevalence of EC and is home to more than half of patients with EC in the world. Majority of EC cases are initially diagnosed at an advanced stage of the disease.^3^ Despite the use of multidisciplinary/multimodal therapies, the 5-year survival rate of patients with EC is only 15%–25%.^4^

Surgery is still the cornerstone of treatment for potentially resectable ESCC. However, among patients with locally advanced EC, the R0 resection rate is low (around 50%), resulting in early recurrence after surgery.^5 6^ The combination of chemotherapy or chemoradiotherapy in the neoadjuvant setting can considerably improve the R0 resection rate and, subsequently, survival.^7^ Although moderately high incidence of pathological response after chemoradiotherapy is reported, the clinical benefit of neoadjuvant therapy in EC is still suboptimal and unsatisfactory. Neoadjuvant chemotherapy increases the R0 resection rate by only 6% and the 5-year survival rate by only 5.9% at most.^6 8^ Studies have shown that, although neoadjuvant chemoradiotherapy can further increase the R0 resection rate, it is associated with more postoperative complications and higher postoperative mortality.^9 10^ A more effective and less toxic neoadjuvant treatment regimen is therefore needed to improve the clinical outcomes of patients with ESCC without increasing the burden of treatment-related adverse events (AEs).

Pembrolizumab and camrelizumab have already shown survival benefit over chemotherapy in the second-line treatment of patients with advanced or metastatic EC.^11 12^ In the KEYNOTE-590 study, pembrolizumab combined with chemotherapy significantly extended overall survival (OS) compared with placebo combined with chemotherapy in the first-line treatment of patients with advanced ESCC (median survival: 12.6 months vs 9.8 months; HR 0.72, 95% CI 0.60 to 0.88), with manageable toxicity.^13^ Immunotherapy has been recommended for treatment of advanced EC by the National Comprehensive Cancer Network guidelines.^14^

Preclinical studies have confirmed that programmed cell death 1 (PD-1) inhibitors combined with chemotherapy can further enhance the host’s immune response and inhibit cancer cell immune escape.^15^ Neoadjuvant treatments combining PD-1 inhibitors with chemotherapy have been shown to induce tumor regression and achieve major pathological response in 83% of patients with lung cancer in the NADIM study.^16^ However, to date, there has been no conclusive evidence to support the effectiveness of neoadjuvant immunotherapy in patients with ESCC.

To lay the foundation for a future randomized clinical trial to demonstrate the clinical efficacy of neoadjuvant PD-1 blockade, we conducted a pilot study to examine the safety and feasibility of using the combination of neoadjuvant PD-1 blockade with chemotherapy in a small group of patients with resectable ESCC. The primary outcomes were safety and feasibility, and the secondary outcomes were objective response rate (ORR), disease control rate (DCR), R0 resection rate, and pathological response rate.

## Methods

### Study design and participants

This investigator-initiated, single-arm, prospective trial of neoadjuvant PD-1 blockade in combination with nab-paclitaxel and carboplatin for resectable ESCC was performed at the First Affiliated Hospital of Sun Yat-sen University. Patient eligibility criteria included the following: (1) aged 18–75 years; (2) clinical stage II–III ESCC as defined by the American Joint Committee on Cancer (AJCC Eighth Edition)^17^ considered to be surgically resectable by a thoracic surgeon; (3) an Eastern Cooperative Oncology Group (ECOG) performance status score of 0–1; (4) adequate organ function; and (5) no prior chemotherapy or radiotherapy. Exclusion criteria included the following: (1) a diagnosis of other malignant tumors within the previous 5 years; (2) history of anti-PD-1 or anti-programmed death-ligand 1 (PD-L1) therapy; (3) history of interstitial lung disease or active non-infectious pneumonia with corticosteroid treatment; and (4) treatment with corticosteroids or other immunosuppressants within the previous 2 weeks.

This Guangdong Association Study of Thoracic Oncology 1056 (GASTO1056) study is registered at http://www.chictr.org.cn/. Written informed consent to participate in the study was obtained from all patients.

### Procedures

Patients received two cycles of drug treatment before surgical resection; in each 21-day cycle, the following were administered intravenously: camrelizumab (200 mg) on day 1, nab-paclitaxel (260 mg/m^2^) on day 1, and carboplatin (area under the curve 5; 5 mg/mL/min) on day 1. At staging and after the first two neoadjuvant treatment cycles, enhanced CT of the neck, chest, and upper abdomen and/or positron emission tomography-CT and ultrasound endoscopy were carried out. Tumor response was assessed by two senior radiologists after two cycles of neoadjuvant treatment and before surgery according to Response Evaluation Criteria in Solid Tumors (RECIST) version 1.1.

Surgery was scheduled for 21–42 days after the first day of the second treatment cycle. Resection of the primary tumor and lymph nodes was performed in line with standard procedures for minimally invasive esophagectomy.^18^ Pathological response was assessed by local pathologists through measurement of the percentage of residual viable tumor after primary tumor resection using previously reported methods.^19–22^ After evaluation, all pathological assessments for response were confirmed by consensus of two blinded pathologists. Pathological complete response (PCR) was defined as the absence of viable tumor cells in the resected cancer specimen; major pathological response (MPR) was defined as the presence of ≤10% viable tumor cells in the resected cancer specimen; pathological partial response (PR) was defined as the presence of >10% but ≤50% viable tumor cells in the resected cancer specimen; pathological stable disease (SD) was defined as the presence of >50% viable tumor cells in the resected cancer specimen; and incomplete pathological response was defined as the presence of >10% viable tumor cells in the resected cancer specimen.

At each visit, patients underwent physical examinations and laboratory tests. AEs and abnormal laboratory findings were assessed according to the National Cancer Institute’s Common Terminology Criteria for Adverse Events (NCI-CTCAE) version 5.0. Treatment was interrupted or delayed if a severe (grade 3–4) AE occurred and would be resumed if protocol-defined criteria for treatment resumption were met. As specified in the trial protocol (online supplemental file 2), in the event of neutropenic fever, prolonged neutropenia, or thrombocytopenia (platelet count of less than 50×10^9^/L), dose reductions for nab-paclitaxel and carboplatin were permitted. Patients had the right to withdraw from the study at any time and for any reason. The investigator had the authority to withdraw patients from the study for unacceptable toxicity, protocol violation, or other reasons.

10.1136/jitc-2021-003497.supp2 Supplementary data

The detailed methodology for follow-up, assessing quality of life, immunohistochemistry, multiplex immunofluorescence staining, and next generation sequencing, including analysis of PD-L1 expression, and CD4^+^, CD8^+^, CD56^+^, PD-1^+^, granzyme B (GRB^+^), T-cell intracellular antigen-1 (TIA-1^+^), and CD163^+^ tumor-infiltrating lymphocytes or macrophages, is described in the Methods section of the online supplemental file 1.

10.1136/jitc-2021-003497.supp1 Supplementary data

### Outcomes

The primary endpoints of this study were safety and feasibility. Toxicity profiles were assessed according to the NCI-CTCAE (version 5.0) guidelines. Surgical outcomes were the operative time (the duration between skin incision and wound closure), intraoperative blood loss, perioperative mortality, and postsurgical complications. The secondary endpoints included MPR, R0 resection rate, ORR, DCR, disease-free survival (calculated from the date of enrollment), and OS. Pretreatment biopsy samples and post-treatment surgical samples were collected to identify immunological and genomic predictors of therapeutic response and to gain a mechanistic insight into the treatment’s efficacy.

### Statistical analysis

Categorical variables were presented as absolute and relative frequencies and numerical variables as mean and SD. Safety data were presented as frequency and percentage of patients affected. Paired Student’s t-test was used for pre–post comparisons and Mann-Whitney U test was used for normally distributed continuous variables and non-normally distributed variables, respectively. The χ^2^ test or Fisher’s exact test was used to analyze the associations between categorical measures and pathological response arms, as appropriate. SAS V.9.4 was used for all statistical analyses, with p<0.05 being considered statistically significant.

## Results

### Overview of patient cohort

**Figure 1:**
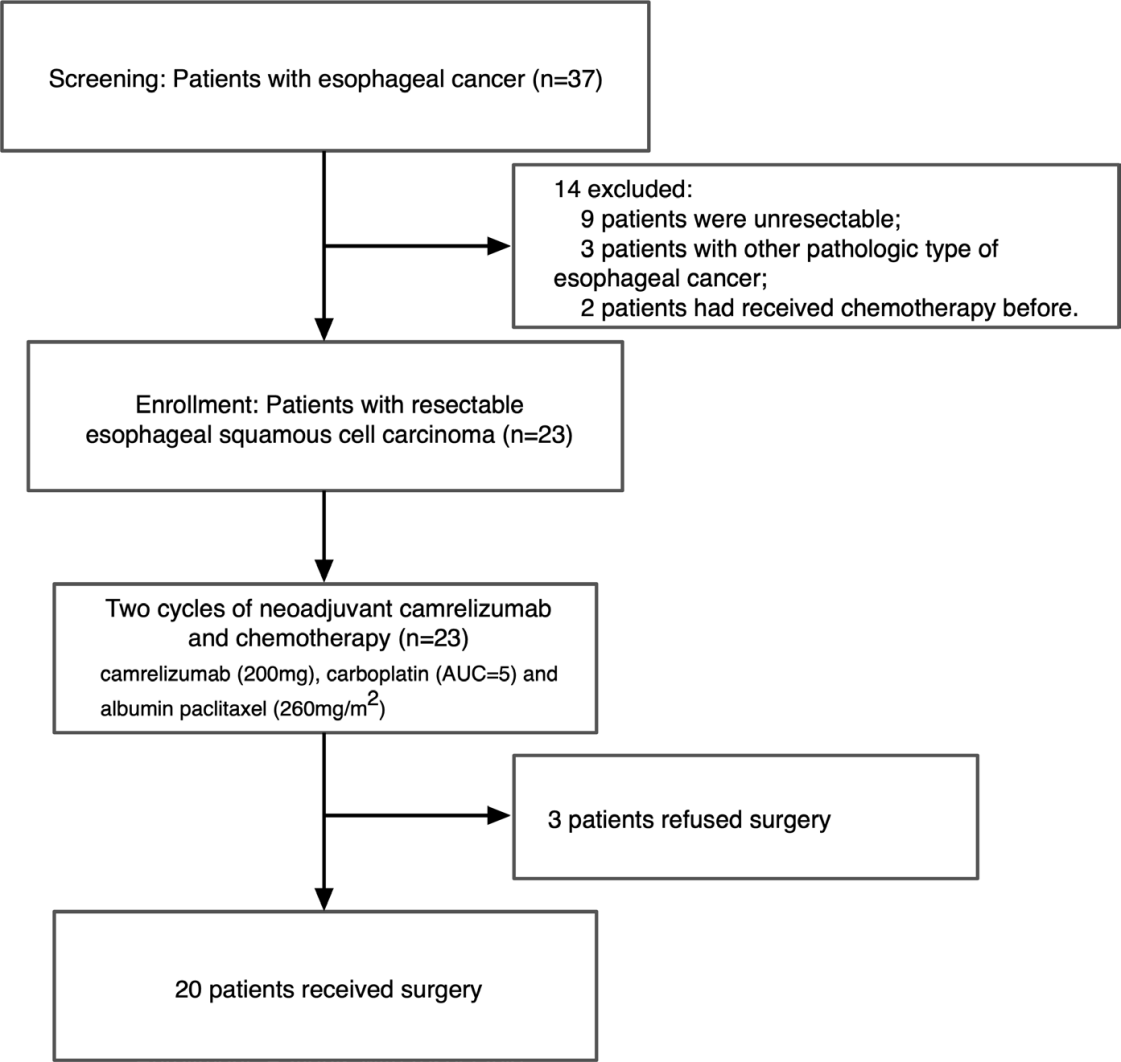
Study flow chart. AUC, area under the curve.

Between January 19, 2020 and September 12, 2020, 37 patients were screened for eligibility; eventually, 23 eligible patients were enrolled after signing informed consent documents (figure 1). All 23 patients finished the two cycles of neoadjuvant therapy, but 3 patients withdrew from the study after refusing surgery. Among the three withdrawn patients, one completed the post-treatment radiological examination before withdrawal. As shown in table 1, the enrolled patients were aged 58.6±10.1 years. Most of the cohort (16 of 23, 69.6%) were smokers, and most patients (22 of 23, 95.7%) were male. The tumor was located in the lower, middle, and upper segment of the esophagus in 13 (56.5%) patients, 9 (39.1%) patients, and 1 (4.3%) patient, respectively. At baseline, 15 (65.2%) patients had AJCC Eighth Edition-defined stage III disease, while the other 8 (34.8%) patients were defined as stage II. Regarding ECOG status, 21 (91.3%) patients had a performance score of 0, and 2 patients had a performance score of 1. PD-L1 expression and tumor mutation burden (TMB) were assessed on pretreatment biopsy samples. A commercially available PD-L1 immunohistochemistry assay (clone 22C3; DAKO Autostainer Link 48; ready to use (RTU)) was used to assess the PD-L1 combined positive score (CPS) according to the manufacturer’s instructions and international guidelines.^23 24^ Samples were considered to be PD-L1-positive if the CPS ≥1. The threshold used to define high TMB (TMB-H) depended on the top 25% of this cohort and the cut-off was 7 Muts/Mb. Due to issues with tissue sample quality, four patients were not evaluated for TMB or PD-L1. Among the 19 samples with available biomarkers, 12 were positive for PD-L1 expression (PD-L1^+^, CPS ≥1) and 5 had TMB-H (≥7 Muts/Mb).

**Table 1:** Baseline characteristics of all enrolled patients

Characteristics	n (%) or mean±SD
Age (years)	58.6±10.1
Gender	
Male	22 (95.7)
Female	1 (4.3)
Smoking status	
Never	7 (30.4)
Former or current	16 (69.6)
Alcohol consumption	
Never	11 (47.8)
Former or current	12 (52.2)
Tumor location	
Upper segment	1 (4.3)
Middle segment	9 (39.1)
Lower segment	13 (56.5)
Clinical TNM stage*	
II	8 (34.8)
III	15 (65.2)
Performance score	
0	21 (91.3)
1	2 (8.7)
PD-L1, CPS	
<1	7 (30.4)
≥1	12 (52.2)
NE	4 (17.4)
TMB status	
TMB-H (≥7 Muts/Mb)	5 (21.7)
TMB-L (<7 Muts/Mb)	14 (60.9)
NE	4 (17.4)

*Clinical disease stage was assessed according to the criteria of the American Joint Committee on Cancer, Eighth Edition.

CPS, combined positive score; NE, not evaluable; PD-L1, programmed death-ligand 1; TMB, tumor mutation burden; TMB-H, tumor mutation burden-high; TMB-L, tumor mutation burden-low; TNM, tumor node metastasis.

### Surgery outcomes

Surgery was performed on 20 patients, all of whom achieved R0 surgical resection (table 2). Minimally invasive esophagectomy (Mckeown) and open esophagectomy were received by 18 (90%) and 2 (10%) patients, respectively. Two patients converted to open surgery due to difficulty in esophageal dissection caused by fibrosis and suspected trachea involvement. The intraoperative blood loss and operative time were 120.0±37.7 mL (mean±SD) and 292.5±53.1 min, respectively. The number of resected lymph nodes and lymph node stations was 29.6±8.8 and 11±1.9, respectively. No treatment-related surgical delays were recorded, and the median interval between the last administration of neoadjuvant therapy and surgery was 31 days (IQR: 24–42). No esophageal fistula attributable to neoadjuvant treatment occurred before surgery. Postoperative complications are summarized in table 2. There were two (10%) cases of anastomotic leakage and one (5%) case each of pulmonary infection, postoperative bleeding, and postoperative hoarseness. No other severe complications such as respiratory failure, heart failure, deep vein thrombosis, or acute respiratory distress syndrome occurred. None of the patients died within 90 days after surgery.

**Table 2:** Surgical and pathological outcomes of patients who underwent surgery

Characteristics	n (%) or mean±SD
Successful R0 resection with curative intent	20 (100)
Surgical approach	
MIE	18 (90.0)
OE	2 (10.0)
Pathological response	
PCR	5 (25.0)
MPR	10 (50.0)
PR	3 (15.0)
SD	2 (10.0)
Downstaging of T stage	
Yes	16 (80.0)
No	4 (20.0)
Downstaging of N stage	
Yes	10 (50.0)
No	10 (50.0)
Downstaging of TNM stage	
Yes	13 (65.0)
No	7 (35.0)
Blood loss (mL)	120.0±37.7
Cumulative operative time (min)	292.5±53.1
Number of resected lymph nodes	29.6±8.8
Number of resected lymph node stations	11±1.9
ICU stay	2 (10.0)
Surgical complications	
Anastomotic leakage	2 (10.0)
Pulmonary infection	1 (5.0)
Postoperative bleeding	1 (5.0)
Postoperative hoarseness	1 (5.0)
90-day mortality	0 (0)

ICU, intensive care unit; MIE, minimally invasive esophagectomy; MPR, major pathological response; OE, open esophagectomy; PCR, pathological complete response; PR, partial response; SD, stable disease; TNM, Tumor Node Metastasis.

### Radiological and pathological response

**Figure 2:**
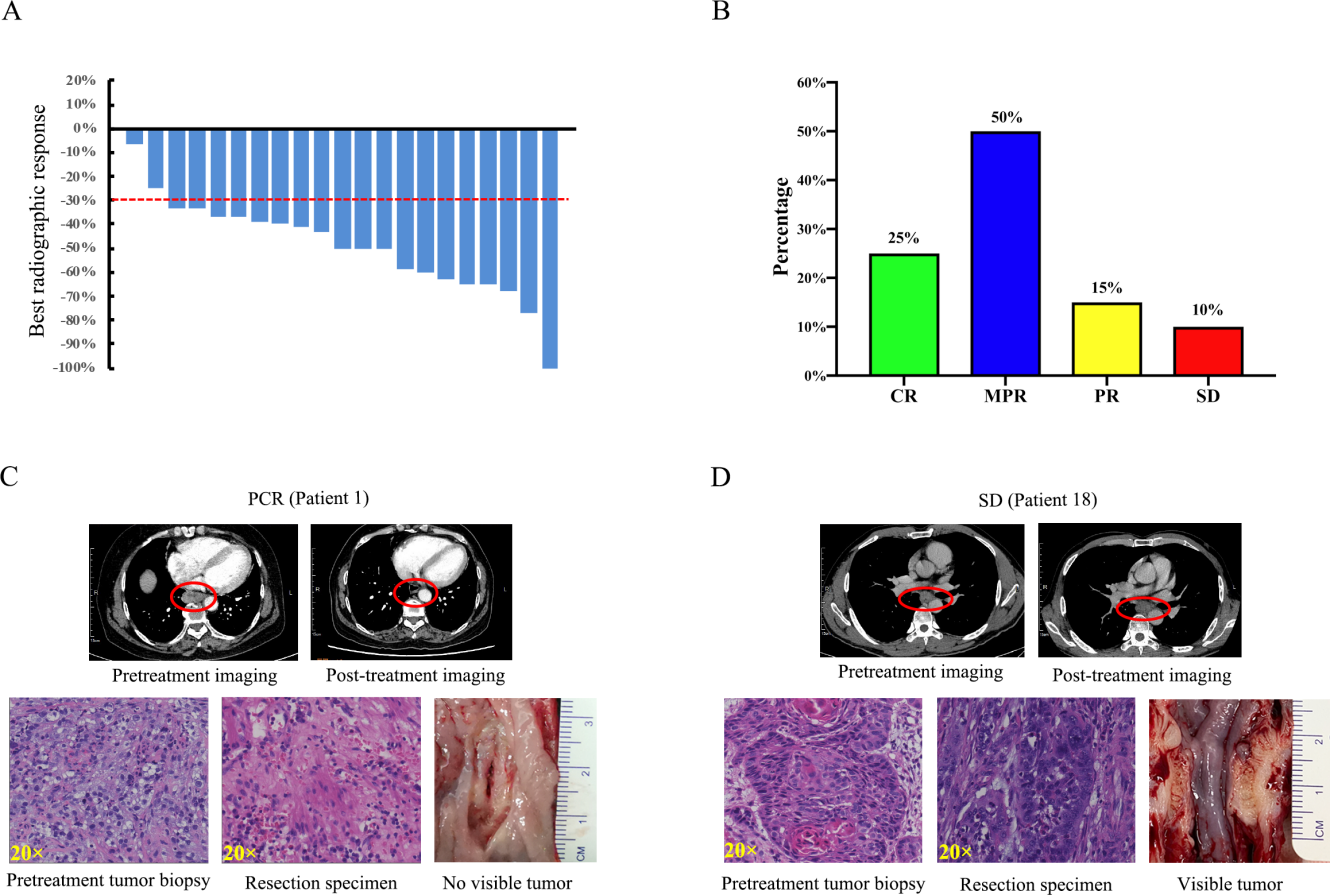
Radiographic and pathological responses to neoadjuvant camrelizumab combined with chemotherapy. (A) Waterfall plots of best radiographic response by RECIST 1.1. (B) Pathological responses of the enrolled patients (n=20) who received surgery. (C) Pretreatment and post-treatment CT and H&E images of a representative patient with a PCR. The esophageal tumor showed significant shrinkage after treatment (red circles). There is no tumor visible in the resected esophagus. (D) Pretreatment and post-treatment CT and H&E images of a representative patient with a pathological response of SD. The esophageal tumor remained stable in size after treatment (red circles). The tumor is still visible in the resected esophagus. CR, complete response; MPR, major pathological response; PCR, pathological complete response; PR, partial response; RECIST, Response Evaluation Criteria in Solid Tumors; SD, stable disease.

According to the RECIST 1.1 criteria, 19 patients who underwent preneoadjuvant and postneoadjuvant therapy imaging attained an objective response: 1 (4.8%) patient had a complete response, 18 (85.7%) patients had PR, while the other 2 (9.5%) patients had SD. No patients had progressive disease during neoadjuvant therapy (figure 2A, online supplemental tables S1 and S2). The ORR and DCR were 90.5% (19 of 21) and 100% (21 of 21), respectively. Of the 20 patients who underwent surgery, 5 (25%) had PCR, 10 (50%) had MPR, 3 (15%) had partial pathological response, and 2 (10%) had SD (figure 2B–D, table 2). Sixteen (80%) patients achieved pathological downstaging of clinical T stage; 10 (50%) patients achieved pathological downstaging of clinical N stage; and 13 (65%) patients achieved pathological downstaging of overall clinical stage (table 2, online supplemental table S3). No significant association was identified between pathological response and smoking status, clinical TNM stage, clinical T stage, or lymph node metastases (online supplemental table S4).

### Treatment-related AEs

All 23 enrolled patients received two cycles of neoadjuvant treatment of camrelizumab plus carboplatin and nab-paclitaxel. Treatment-related AEs are summarized in table 3. The most frequently occurring treatment-related AE of any grade was alopecia, which occurred in 19 (82.6%) of the 23 patients. Asthenia (15 of 23, 65.2%), neutropenia (14 of 23, 60.9%), leukopenia (14 of 23, 60.9%), rash (14 of 23, 60.9%), anemia (12 of 23, 56.5%), and increased alanine aminotransferase (10 of 23, 43.5%) were also common among the patients. Despite the high incidence of reactive cutaneous capillary endothelial proliferation, which is commonly associated with camrelizumab, only cases of grade 1 or 2 were recorded (9 patients, 39.1%). The most common grade 3–4 AEs were neutropenia (9 of 23, 39.1%) and leukopenia (2 of 23, 8.7%). None of the AEs reported during neoadjuvant treatment led to discontinuation of treatment, dose reduction, or surgical delay. No treatment-related mortality occurred.

**Table 3:** Neoadjuvant treatment-related adverse events

	Any grade	Grades 1–2	Grade 3	Grade 4
Neutropenia	14 (60.9)	5 (21.7)	5 (21.7)	4 (17.4)
Leukopenia	14 (60.9)	12 (52.2)	1 (4.3)	1 (4.3)
Alopecia	19 (82.6)	19 (82.6)	0	0
Asthenia	15 (65.2)	15 (65.2)	0	0
Rash	14 (60.9)	14 (60.9)	0	0
Anemia	13 (56.5)	13 (56.5)	0	0
Alanine aminotransferase increased	10 (43.5)	10 (43.5)	0	0
Aspartate aminotransferase increased	8 (34.8)	8 (34.8)	0	0
Reactive cutaneous capillary endothelial proliferation	9 (39.1)	9 (39.1)	0	0
Hyperbilirubinemia	8 (34.8)	8 (34.8)	0	0
Decreased appetite	8 (34.8)	8 (34.8)	0	0
Thrombocytopenia	7 (30.4)	7 (30.4)	0	0
Vomiting	5 (21.7)	5 (21.7)	0	0
Oral mucositis	4 (17.4)	4 (17.4)	0	0
Nausea	3 (13.0)	3 (13.0)	0	0
Diarrhea	3 (13.0)	3 (13.0)	0	0
Constipation	3 (13.0)	3 (13.0)	0	0
Edema	2 (8.7)	2 (8.7)	0	0
Fever	2 (8.7)	2 (8.7)	0	0
Hyperthyroidism	1 (4.3)	1 (4.3)	0	0
Arthralgia	1 (4.3)	1 (4.3)	0	0
Peripheral sensory neuropathy	1 (4.3)	1 (4.3)	0	0

Data are presented as n (%).

Adverse events were graded according to the National Cancer Institute’s Common Terminology Criteria for Adverse Events, version 5.0.

### Quality of life

Health-related quality of life was assessed and compared between baseline and postneoadjuvant therapy using the European Organization for Research and Treatment of Cancer’s Quality of Life Questionnaire-Core 30 and the Quality of Life Questionnaire-Esophageal Cancer Module-18. Overall quality of life increased significantly (p<0.0001) from baseline to postneoadjuvant therapy. Patients’ physical (p=0.0244), emotional (p=0.0200), and cognitive (p=0.0158) functioning increased at post-treatment assessment compared with baseline. After the neoadjuvant therapy, fatigue (p=0.008), nausea and vomiting (p=0.0018), pain (p=0.0001), appetite loss (p=0.0153), and financial difficulties (p=0.0237) were alleviated, but there was no significant difference in the other aspects assessed by the questionnaires. Compared with those at baseline, symptoms of dysphagia (p=0.0002), difficulty swallowing saliva (p=0.0493), choking when swallowing (p<0.0001), eating (p=0.0001), and pain (p=0.0014) were significantly alleviated after neoadjuvant therapy (online supplemental table S5).

### Follow-up

Up to June 30, 2021, the median follow-up was 13.77 months (IQR: 9.7–17.6) from the first day of treatment. During follow-up, 5 (25%) of the 20 patients who received surgery experienced disease recurrence or metastasis ranging from 4 to 12 months after surgery. None of them was found to have recurrence or metastasis on routine CT scan at 3 months after surgery. The pathological response of three patients was MPR and that of the other two patients was PR. Among these five patients, one had recurrence in the supraclavicular lymph nodes and liver metastasis at 6 months after surgery, one had disease recurrence in the mediastinal lymph nodes and liver metastasis at 4 months after surgery, and the other three had disease recurrence in the mediastinal lymph nodes at 8, 6, and 12 months after surgery, respectively (online supplemental table S3).

**Figure 3:**
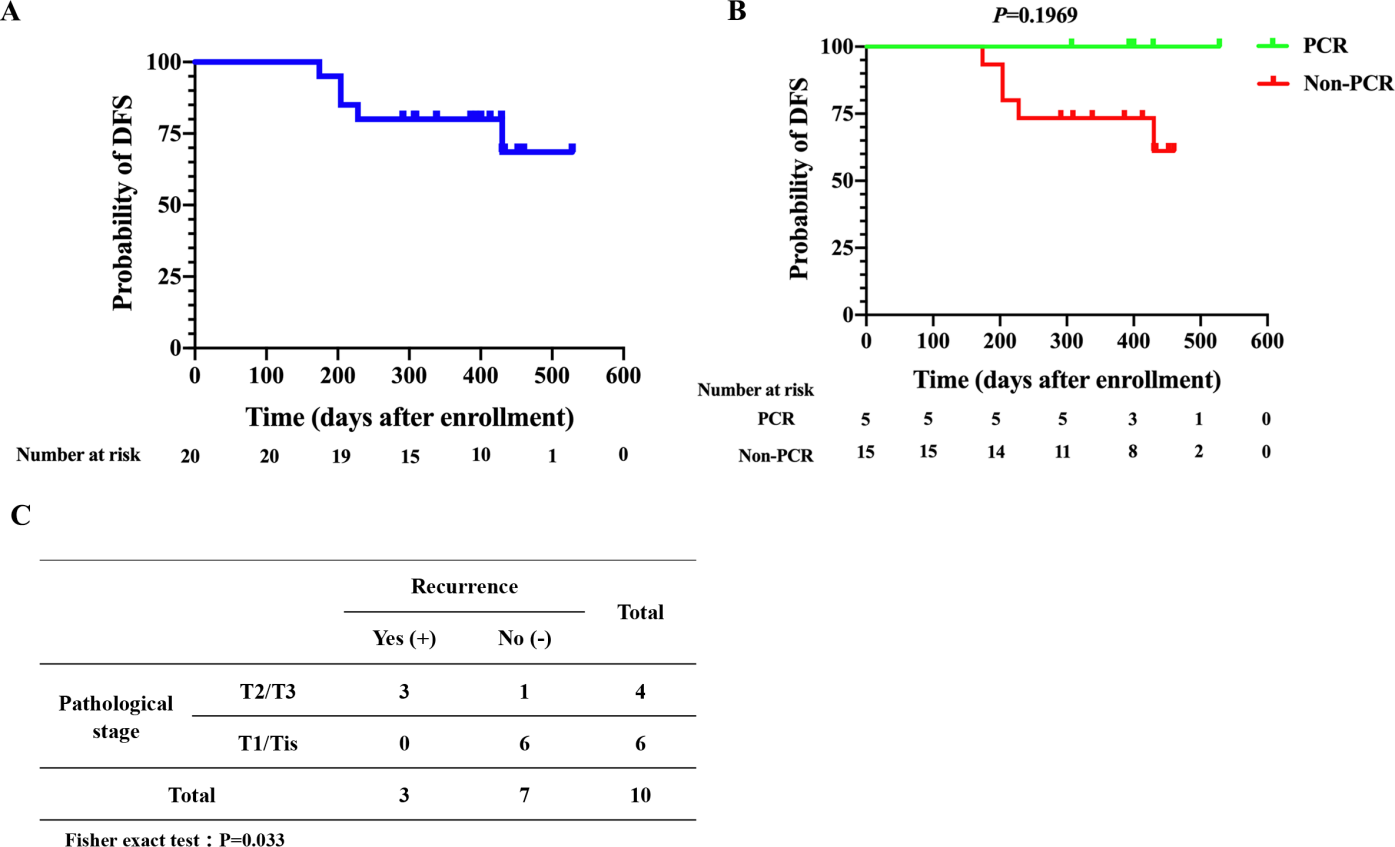
DFS curves of patients who received surgery (n=20). (A) DFS curve of all patients who received surgery (n=20). (B) DFS curves of the PCR group (n=5) and the non-PCR group (n=15). (C) Comparison of pathological T stage and recurrence among MPR patients (n=10) in a 2×2 contingency table. DFS, disease-free survival; MPR, major pathological response; PCR, pathological complete response.

In the entire cohort of patients who received surgery, the median disease-free survival was not reached (figure 3A). For patients who achieved PCR, there was no significantly improved disease-free survival over those without PCR (figure 3B). Of note, there may be some potential confounders in the survival analysis such as comorbidity and concurrent medications that cannot be minimized due to the small sample size. Among the 10 patients with MPR, those with a pathological stage of T2/T3 had a higher risk of tumor recurrence or metastases than those with T1/Tis disease (p=0.033; figure 3C).

### Immunohistochemistry and multiplex immunofluorescence staining

**Figure 4:**
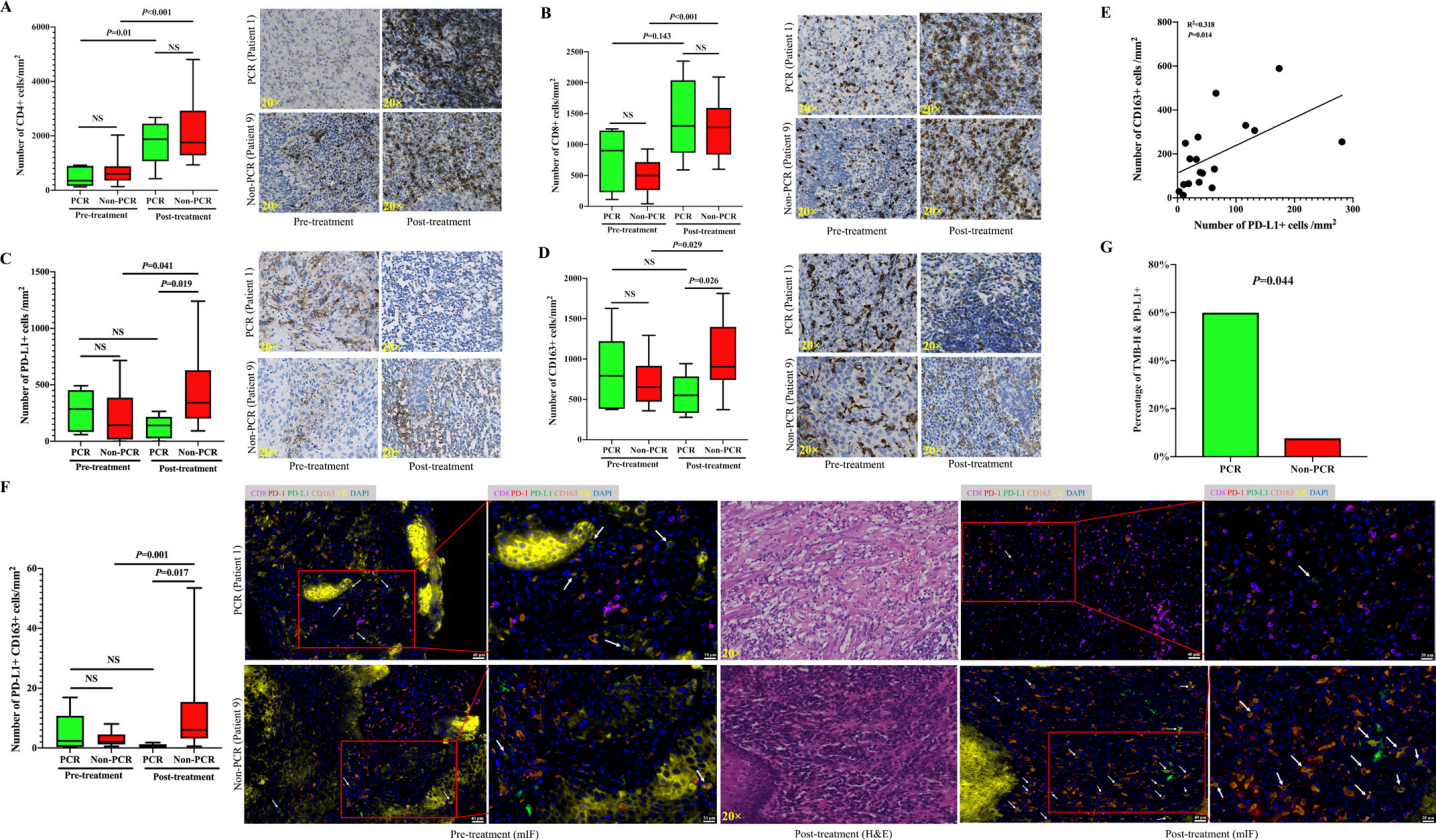
The immune microenvironment is correlated with the response to neoadjuvant camrelizumab combined with chemotherapy. (A) Comparison of infiltrating CD4^+^ cells between the PCR group (n=5) and the non-PCR group (n=14) before and after treatment. (B) Comparison of infiltrating CD8^+^ cells between the PCR group (n=5) and the non-PCR group (n=14) before and after treatment. (C) Comparison of infiltrating PD-L1^+^ cells between the PCR group (n=5) and the non-PCR group (n=14) before and after treatment. (D) Comparison of infiltrating CD163^+^ cells between the PCR group (n=5) and the non-PCR group (n=14) before and after treatment. (E) Correlation between infiltrating PD-L1^+^ and CD163^+^ cells in post-treatment samples based on multiplex immunofluorescence staining (n=18). (F) Comparison of change in PD-L1^+^ CD163^+^ cells between the PCR group (n=5) and the non-PCR group (n=13) before and after treatment based on multiplex immunofluorescence staining. A significant increase in PD-L1^+^ CD163^+^ cells (white arrows) is observed after neoadjuvant chemoimmunotherapy in the non-PCR group. Antibody panel: CD8 (magenta), PD-1 (red), PD-L1 (green), CD163 (orange), cytokeratin (CK, yellow), and 2-(4-amidinophenyl)-6-indolecarbamidine dihydrochloride (DAPI, blue). (G) The percentage of patients with both TMB-H and PD-L1^+^ was significantly higher in the PCR group (n=5) than those in the non-PCR group (n=14). mIF, multiplex immunofluorescence; PCR, pathological complete response; PD-L1, programmed death-ligand 1; TMB-H, tumor mutation burden-high.

To examine the immune microenvironment and its potential association with pathological response, we performed immunohistochemistry to detect PD-L1 and other immune biomarkers in paired pretreatment tumor biopsies and post-treatment surgical resections obtained from 19 of the patients. The calculation of immune cells was performed both in the stromal region and in the tumor region in non-PCR patients. For cases with PCR after therapy, only stromal regions were scored due to no residual viable tumor cells. No significant difference was observed in the expression of PD-L1 determined by CPS between patients with PCR and those without PCR (online supplemental figure S7C). Significant increases in the number of infiltrating CD4^+^, CD8^+^, CD56^+^, PD-1^+^, GRB^+^, and TIA-1^+^ cells were observed after neoadjuvant chemoimmunotherapy, but there was no significant change in the number of infiltrating PD-L1^+^ and CD163^+^ cells (online supplemental figure S1). Increases in the number of infiltrating CD4^+^, CD8^+^, CD56^+^, PD-1^+^, GRB^+^, and TIA-1^+^ cells were observed in both the PCR and the non-PCR groups after treatment. There was no significant difference in the number of infiltrating CD4^+^, CD8^+^, CD56^+^, PD-1^+^, GRB^+^, or TIA-1^+^ cells in the pretreatment and post-treatment samples between the PCR and non-PCR groups (figure 4A, B, online supplemental figure S2). Similar trends of the infiltrating CD4^+^, CD8^+^, CD56^+^, PD-1^+^, GRB^+^, and TIA-1^+^ cells were observed between the PCR +MPR and PR+SD groups (online supplemental figure S3). Of note, after treatment, there were far more infiltrating PD-L1^+^ and CD163^+^ cells in the non-PCR group than in the PCR group (figure 4C, D); moreover, in the non-PCR group, the number of infiltrating PD-L1^+^ and CD163^+^ cells was significantly increased after treatment compared with before treatment (figure 4C, D). There was no significant difference in infiltrating CD163^+^ cells in the pretreatment and post-treatment samples between the PCR +MPR and PR+SD groups (online supplemental figure S3). The change in infiltrating immune cells after treatment in each patient was calculated by the density of infiltrating immune cells in the post-treatment samples divided by the infiltrating immune cells in the pretreatment samples. After treatment, PD-L1^+^ and CD163^+^ cells tended to show an increased number from the pretreatment in the non-PCR group, but the opposite tendency was seen in the PCR group (online supplemental figure S4). There was no significant difference between changes in the pretreatment and post-treatment number of infiltrating CD4^+^, CD8^+^, CD56^+^, PD-1^+^, GRB^+^, and TIA-1^+^ cells between the PCR and non-PCR groups (online supplemental figure S4). Based on these findings, we further examined multiplexed immunofluorescence using the antibodies for CD8, CD163, PD-1, PD-L1, and cytokeratin (CK) to characterize the immune microenvironment of the tumor. CK was used to define the tumor region, and the density of markers in the CK-positive tumor region and stromal region was evaluated separately. The results showed that the number of PD-L1^+^ cells was positively correlated with CD163^+^ cells and PD-L1^+^ CD163^+^ cells at pretreatment and post-treatment, respectively (figure 4E, online supplemental figure S5). However, there was no correlation between PD-L1^+^ cells and infiltrating CD8^+^, PD-1^+^, or CD8^+^ PD-1^+^ cells (online supplemental figure S5). Furthermore, we found that the number of infiltrating PD-L1^+^ CD163^+^ cells in the non-PCR group was significantly higher than in the PCR group after neoadjuvant treatment, and the number of PD-L1^+^ CD163^+^ cells was significantly increased after treatment compared with before treatment in the non-PCR group (figure 4F, online supplemental figure S6). Additionally, there was no significant difference in the number of infiltrating CD8^+^ PD-1^+^ cells in the pretreatment and post-treatment samples between the PCR +MPR and PR+SD groups (online supplemental figure S3) or the PCR and non-PCR groups (online supplemental figure S6).

### Genomic analyses

We further performed next generation sequencing of pretreatment tumor specimens obtained from 19 patients who had adequate amounts of tissues available. A median of 10 somatic mutations (range: 2–32) per tumor was noted, and specific driver mutations identified included TP53, CDKN2A, CDKN2B, CCND1, and MYC (online supplemental figure S7A). Patients with PCR demonstrated a higher TMB compared with patients without PCR, but it was not statistically significant (p=0.083; online supplemental figure S7B). The percentage of patients with both TMB-H and PD-L1^+^ was significantly higher in the PCR group (p=0.044; figure 4G). No significant difference in the immune-related pathways was found between the PCR and non-PCR groups (online supplemental figure S7D). No significant difference was found in disease-free survival based on different TMB and PD-L1 status (online supplemental figure S8). There was also no significant difference in PD-L1 and TMB-H status between downstaged and non-downstaged patients (online supplemental table S6).

## Discussion

Our study reported the application of neoadjuvant PD-1 blockade in combination with chemotherapy in patients with resectable (stage II or III) ESCC. Neoadjuvant camrelizumab plus carboplatin and nab-paclitaxel had manageable treatment-related toxic effects and did not delay surgery. This regimen induced PCR or MPR in 75.0% of resected tumors, demonstrating its antitumor efficacy in resectable ESCC.

Overall, the neoadjuvant combination therapy of camrelizumab with carboplatin and nab-paclitaxel had favorable safety and feasibility. In terms of toxicity, the main treatment-related AE of grade 3–4 was neutropenia (39.1%), the incidence of which was lower than those reported in the MRC OE02 neoadjuvant chemotherapy group (61.3%) and the NEOCRTEC5010 neoadjuvant chemoradiotherapy group (48.8%).^6 25^ In our study, the incidence of reactive cutaneous capillary endothelial proliferation, an AE commonly associated with camrelizumab, was 39.1%, which was much lower than the incidence reported for camrelizumab as an advanced second-line therapy (79%).^12^ Therefore, camrelizumab combined with chemotherapy does not appear to increase side effects as a therapy for EC in the neoadjuvant setting.

In terms of surgical safety, the neoadjuvant therapy in this study did not delay surgery and the R0 resection rate reached 100%, while in previous studies the reported R0 resection rates with neoadjuvant chemotherapy and neoadjuvant chemoradiotherapy were 60% and 98%, respectively.^6 25^ Moreover, the average number of resected lymph nodes (29.6) was significantly higher than those reported in the CROSS (15.0) and NEOCRTEC5010 (20.0) studies.^25 26^ These results demonstrate that with this neoadjuvant therapy the R0 resection rate was high, and it did not increase the difficulty of achieving complete resection of the primary tumor or lymph nodes. In terms of postoperative complications, anastomotic leakage (10%) had the highest incidence in this study, which was lower than the incidence previously reported for neoadjuvant chemoradiotherapy (23.1%).^27^ Moreover, no perioperative deaths occurred in this study. Collectively, these results suggest that the toxicity of neoadjuvant immunotherapy combined with camrelizumab, carboplatin, and nab-paclitaxel is acceptable.

Encouragingly, in this study, the PCR rate of neoadjuvant therapy with camrelizumab combined with carboplatin and nab-paclitaxel reached 25%, which was higher than that previously reported for neoadjuvant chemotherapy (10.2%)^28^ and similar to that previously reported for neoadjuvant PD-1 blockade in combination with chemotherapy (33%).^29^ Previous studies have shown that achieving an MPR after neoadjuvant therapy is associated with a better survival outcome in other cancers, such as lung cancer.^30^ In patients who achieved an MPR, we found that those with a pathological stage of T2/T3 had a higher risk of tumor recurrence or metastasis than those with T1/Tis, indicating that different pathological T stages may lead to different prognoses among patients with MPR after neoadjuvant immunochemotherapy. Among the 20 surgical patients, 13 (65%) achieved downstaging after treatment, which was higher than reported in the previous literature (40%).^28^ Previous studies have suggested that patients with EC who achieve downstaging after neoadjuvant therapy may have a better survival outcome.^31^ Our data also showed that patients’ quality of life was significantly improved after neoadjuvant therapy and their symptoms of dysphagia were significantly relieved, which might be related to the high PCR and downstaging rates. These encouraging results provide clinical evidence for the application of immunotherapy combined with chemotherapy in the neoadjuvant setting.

The tumor immune microenvironment of ESCC has been reported to be in an immunosuppressive state dominated by exhausted T and natural killer (NK) cells.^32^ In the present study, there were few tumor-infiltrating immune cells before treatment; however, a significant increase in tumor-infiltrating CD4^+^, CD8^+^, and CD56^+^ lymphocytes was observed after therapy. The priming of CD4^+^ and CD8^+^ T cells helps signals to cytotoxic T lymphocytes and further establishes efficient and durable anti-tumor immunity.^33 34^ CD56^+^ cells are a major cell subset of NK cells, which provide protection against infectious pathogens and cancer.^35^ Our findings suggest that neoadjuvant PD-1 blockade might enhance the systemic priming of antitumor T cells and natural killer cells in the ESCC microenvironment. However, the number of infiltrating PD-L1^+^ CD163^+^ cells significantly increased in the non-PCR group after therapy. It is well-known that CD163 is a specific biomarker of M2-like macrophages, and it was reported that M2-like macrophages with increased expression of PD-L1 could promote immunosuppression. 36–38 Our findings suggest that the induction of M2-like macrophages with increased expression of PD-L1 may be associated with ineffective immunotherapy. The association between changes in the tumor immune microenvironment and the efficacy of neoadjuvant chemoimmunotherapy in ESCC needs to be further verified in full-stage studies. The PD-L1 expression level and TMB are the most studied predictive markers of the efficacy of immune checkpoint inhibitors in the ESCC clinical trial of pembrolizumab (KEYNOTE-181 and KEYNOTE-590).^11 13^ In our study, the percentage of patients with both TMB-H and PD-L1^+^ was significantly higher in the PCR group, suggesting that neoadjuvant chemoimmunotherapy may favor patients with both high genomic instability and PD-L1 expression. The prognostic value of TMB and PD-L1 in patients receiving this regimen should be further verified by larger-scale clinical studies.

There are some limitations to this study. First, due to this study being an exploratory pilot study, the number of enrolled patients was small. Therefore, our findings and the survival data need to be interpreted with caution since some potential confounders may significantly influence the results, and full-scale randomized controlled trials are required to further verify our findings. Second, the follow-up time was short and the median survival was not reached. Longer follow-ups are needed to examine whether neoadjuvant immunochemotherapy can deliver long-term survival benefits for patients. Further investigation into the optimal duration of treatment and biomarkers to predict response should be a focus of future research.

In summary, we report that neoadjuvant camrelizumab plus carboplatin and nab-paclitaxel has good safety and feasibility and does not delay surgery. This regimen has favorable antitumor efficacy. Neoadjuvant camrelizumab combined with carboplatin and nab-paclitaxel is a potential treatment strategy for ESCC. However, the impact of adjuvant anti-PD-1 therapy remains to be examined.

## Data Availability

Data are available upon reasonable request. The data sets generated in the current study are available from the corresponding author on reasonable request.
