# Supplementary material for: Neoadjuvant programmed cell death 1 blockade combined with chemotherapy for resectable esophageal squamous cell carcinoma
Source: J Immunother Cancer. 2022 Jan 12;10(1):e003497. doi: 10.1136/jitc-2021-003497 (PMC8756283; doi:10.1136/jitc-2021-003497)
Supplement: Supplementary data [file jitc-2021-003497supp002.pdf]

A prospective, single-arm clinical study on the safety and efficacy of camrelizumab combined with carboplatin and nab-paclitaxel in the neoadjuvant therapy of potentially resectable stage II-III esophageal squamous cell carcinoma

Protocol

Project No.: GASTO-1056

Version number: Version 1.0

Version date: December 1, 2019

Center: The First Affiliated Hospital of Sun Yat-sen University

Principal Investigator: Chao Cheng

58 Zhongshan 2nd Road, Guangzhou 510080, P. R. China

Phone: +86-20-87755766-8782

E-mail: chengch3@mail.sysu.edu.cn

Contents

Protocol Synopsis 4
1.Background and rationale 16
1.1 Epidemiology of esophageal cancer 16
1.2 Efficacy and deficiency of neoadjuvant chemotherapy in the treatment of stage II-III esophageal cancer 16
1.3 Neoadjuvant chemotherapy and neoadjuvant chemoradiotherapy. 17
1.4 Immunotherapy for esophageal cancer 17
1.5 Rationale for immunotherapy combined with chemotherapy for resectable esophageal squamous cell carcinoma 18
1.6 References 19
2. Objectives 21
2.1 Primary 21
2.2 Secondary 21
3. Study design 22
4. Patient selection 23
4.1 Inclusion Criteria 23
4.2 Exclusion Criteria 24
4.3 Withdraw criteria 27
5. Treatment Plan 28
5.1 Neoadjuvant administration Regimen 28
5.2 Surgical plan and Procedure: 28
6. Dose adjustment 30
6.1 Dose modification of camrelizumab 30
6.2 Dose modification of nab-paclitaxel 32
6.3 Dose modification of carboplatin 33
7. Concomitant medication 34
7.1 Drugs cannot be used during treatment 34
7.2 Concomitant therapy during treatment 34
8.Study process 34
9. Adverse events 36
9.1 Adverse events include but are not limited to 36

9.2 Criteria for the severity of adverse events 37
9.3 Recording and management of adverse events 37
9.4 Serious adverse events 38
9.5 Safety Evaluation 38
9.6 Management of tumor recurrence and metastasis 38
10.Others 39
10.1 Case Report 39
10.2 Ethical Requirements 39
10.3 Quality control 39
10.4 Training of researchers 39
10.5 Improve the compliance of patients 40
10.6 Storage and summary of data 40
11. Attachments 41
Annex 1: TNM staging Criteria for Esophageal cancer (AJCC 8th Edition) 41
Annex 2: ECOG PS Scoring Criteria 41
Annex 3: Response Evaluation Criteria in Solid Tumors-version 1.1 (RECIST V1.1) 41
Annex 4: Evaluation criteria for Common Terminology Criteria for Adverse Events 5.0 (CTCAE V 5.0) 41
Annex 5: NCCN Guidelines for the Management of Immunotherapy-associated Toxicities (2019 V1.0) 41
Annex 6: List of pre-inclusion autoimmune diseases 41
Annex 7: Guidelines for dose adjustment and toxicity management of camrelizumab 41
Annex 8: Quality of Life Scale of EORTC QLQ-C30 and EORTC QLQ-OES18 41

Protocol Synopsis

Objective	Primary objective：<br><br>(1) To evaluate the safety and feasibility of camrelizumab combined with carboplatin and nab-paclitaxel in the neoadjuvant therapy of potentially resectable stage II-III esophageal squamous cell carcinoma.<br><br>Secondary objectives：<br><br>(1) To evaluate the efficacy of camrelizumab combined with carboplatin and nab-paclitaxel in neoadjuvant therapy of potentially resectable stage II-III esophageal squamous cell carcinoma (objective response rate, ORR; disease control rate DCR; major pathological response rate MPR, R0 resection rate, etc.).<br><br>(2) To evaluate the effect of camrelizumab combined with carboplatin and nab-paclitaxel on quality of life of patients undergoing neoadjuvant therapy for stage II-III potentially resectable esophageal squamous cell carcinoma.<br><br>(3) To evaluate the disease-free survival (DFS) of patients receiving neoadjuvant therapy of camrelizumab combined with carboplatin and nab-paclitaxel for stage II-III potentially resectable esophageal squamous cell carcinoma.<br><br>(4) To evaluate the overall survival (OS) of patients receiving neoadjuvant therapy of camrelizumab combined with carboplatin and nab-paclitaxel for stage II-III potentially resectable esophageal squamous cell carcinoma.<br><br>(5) To evaluate the correlation between immune-related markers (PD-L1, tumor mutation burden TMB, tumor neoantigen burden TNB,

	microsatellite instability, MSI, tumor-infiltrating lymphocyte TIL, DDR pathway, etc.) and the efficacy of camrelizumab in neoadjuvant therapy for stage II-III potentially resectable esophageal squamous cell carcinoma.
Study design	Single-center, prospective, single-arm clinical study
Sample size	20 patients
Patients	Patients with clinical TNM stage II-III potentially resectable esophageal squamous cell carcinoma
Inclusion criteria	1. The patient's pathological biopsy results must be confirmed to be esophageal squamous cell carcinoma (not including mixed adenosquamous carcinoma or other pathological types) by pathologists.<br>2. Age ≥18 years old and ≤75 years old.<br>3. ECOG or PS score is 0 or 1.<br>4. Patients did not previously receive cancer related chemotherapy, radiotherapy, or surgery.<br>5. Endoscopically diagnosed cervical esophageal cancer and gastroesophageal junction tumors were not included in this study.<br>6. Clinical stage should be T2-3/N0-2/M0 (II-III), with potential of radical surgical treatment.<br>7. Patients should have PET-CT examination or neck/chest and upper abdomen CT scan, ECT, brain MR/CT, etc. to clarify staging and exclude distant metastasis.<br>8. Adequate organ and marrow function as defined below:<br>1) Blood routine examination: Absolute neutrophil count (ANC)

Yang W, *et al. J Immunother Cancer* 2022; 10:e003497. doi: 10.1136/jitc-2021-003497

	≥1.5×109/L; Platelet count (PLT) ≥100×109/L; Hemoglobin content (HGB) ≥ 9.0g /dL.<br><br>2) Liver function: serum total bilirubin (TBIL) ≤1.5×Upper Limit of Normal (ULN); Alanine aminotransferase (ALT) and aspartate aminotransferase (AST) ≤2.5×ULN.<br><br>3) Renal function: Creatinine clearance (Ccr) ≥60 mL/min (calculated by Cockcroft/Gault formula):<br><br>$$\text{female：Ccr} = \frac{(140 - \text{age}) \times \text{weight(kg)} \times 0.85}{72 \times \text{Scr}\left(\frac{\text{mg}}{\text{dL}}\right)}$$<br>$$\text{male：Ccr} = \frac{(140 - \text{age}) \times \text{weight(kg)} \times 1.00}{72 \times \text{Scr(mg/dL)}}$$<br><br>4) Adequate coagulation function, defined as international normalized ratio (INR) or prothrombin time (PT) ≤1.5 ULN; If patient is receiving anticoagulant therapy, it is acceptable that PT is in the prescribed anticoagulant range.<br><br>9. Female patients of reproductive age or male patients whose sexual partner is female patients of reproductive age shall use effective contraceptive measures throughout the treatment period until 6 months after the treatment period.<br><br>10. Sign written informed consent and be able to comply with the visits and related procedures specified in the program.<br><br>11. Can provide archived pathological tissues or fresh pathological tissues within 6 months for the detection of PD-L1 and other markers.
Exclusion criteria	1. Patients were taking other investigational drugs at the same time.<br><br>2. Those who have not recovered from recent major surgical procedure.

	3.Any treatment with anti-PD-1, anti-PD-L1, anti-PD-L2, anti-CD137, or anti-CTLA-4 antibodies, or any other antibody or drug that specifically targets at T-cell costimulation or checkpoint pathways prior to study.<br>4. History of allergic reactions attributed to any monoclonal antibody or chemotherapy drug (paclitaxel, carboplatin) preparations or excipients.<br>5. Patients were also taking rifampicin, phenytoin sodium, carbamazepine, or barbiturates (these drugs induce CYP3A and may reduce plasma content of paclitaxel).<br>6. Received systemic therapy of Chinese herbal medicine with anti-tumor indications or immunomodulatory drugs (including thymosin, interferon, interleukin, etc.) within 2 weeks prior to first administration.<br>7. Administration of a live, attenuated vaccine within 4 weeks prior to the first dose of treatment or planned for it during the study.<br>Note: Administration of inactivated virus vaccine for seasonal influenza is permitted within 4 weeks prior to the first dose of treatment， while live attenuated flu vaccines are not allowed.<br>8. Toxicity from prior antineoplastic therapy that did not return to grade 0 or 1 defined by National Cancer Institute General Adverse Event Terminology version 5.0 (NCI CTCAE version 5.0, excluding alopecia, non-clinically significant or asymptomatic laboratory abnormalities).<br>9. Known autoimmune disease that needs symptomatic treatment or history of disease within 2 years (patients with vitiligo, psoriasis, hair loss, or graves disease that doesn’t need systemic treatment, hypothyroidism that only needs thyroid hormone replacement therapy and type 1 diabetes

	which only need insulin replacement therapy can be enrolled).<br><br>10. Known history of primary immunodeficiency.<br><br>11. Known to have active infection of tuberculosis.<br><br>12. Known history of allogeneic organ transplantation and allogeneic hematopoietic stem cell transplantation.<br><br>13. HIV infection and carriers are known to exist (HIV antibody positive).<br><br>14. Severe infections that are in active period or clinically poorly controlled.<br><br>15. Symptomatic congestive heart failure (NYHA II-IV) or symptomatic or poorly controlled arrhythmia.<br><br>16. Uncontrolled arterial hypertension (systolic blood pressure ≥160mmHg or diastolic blood pressure ≥100mmHg) even with standard therapy.<br><br>17. Any arterial thromboembolic event, including myocardial infarction, unstable angina, cardiocerebral events or transient ischemic attack (TIA), occurred within 6 months prior to enrollment.<br><br>18. Significant malnutrition which need intravenous supplements while malnutrition corrected for over 4 weeks prior to first dose of the study was excluded.<br><br>19. A history of deep vein thrombosis, pulmonary embolism, or any other severe thromboembolism within 3 months prior to enrollment (Implantable Venous Access Port or duct-derived thrombosis, or superficial venous thrombosis is not considered as "severe" thromboembolism).

	20. Uncontrolled metabolic disorders or other non-malignant organ or systemic diseases or secondary reactions to cancer that may result in higher medical risk and/or uncertainty in the assessment of survival.<br>21. Hepatic encephalopathy, hepatorenal syndrome，Child-Pugh B liver cirrhosis or worse.<br>22. History of intestinal obstruction or following diseases: inflammatory bowel disease (IBD) or extensive bowel resection (partial resection of the colon or extensive resection of the small intestine with chronic diarrhea), Crohn's disease, ulcerative colitis (UC).<br>23. Known to have acute or chronic active hepatitis B (HBsAg positive with HBV DNA viral load $\geq 10^3$ IU/ml /mL or >200IU/ mL) or acute or chronic active hepatitis C (HCV antibody positive and HCV RNA positive).<br>24. History of gastrointestinal perforation and/or fistula within 6 months prior to enrollment.<br>25. Interstitial lung disease (ILD) requiring steroid therapy.<br>26. History of other primary malignancies, excluding:<br>1) Complete remission (CR) of malignant tumors for at least 2 years before enrollment and no other treatment was required during the study;<br>2) Non-melanoma skin cancer or Lentigo maligna (LM) that has been adequately treated and has no evidence of disease recurrence;<br>3) Adequately treated carcinoma in situ (CIS) with no evidence of disease recurrence.

	27. Pregnant or lactating female patients.<br><br>28. Other acute or chronic diseases, psychiatric disorders, or abnormal laboratory test values that may cause： increase related risk of study participation or drug administration, interfere with the interpretation of study results or ineligible patients for study participation from researcher's judgment.<br><br>29. Patients with difficulty in obtaining satisfactory biopsy specimens through endoscopy for relevant detection of the study.
Withdraw criteria	1. The patient is found to be ineligible for the inclusion/exclusion criteria and is deemed unsuitable for further study by the investigator.<br><br>2. The patient violates the study protocol, and it is considered inappropriate to continue to participate in further study by the investigator.<br><br>3. Any other type of pharmaceutical research that is considered scientifically or medically incompatible with this study.<br><br>4. The patient fails to finish the defined follow-up evaluations (Research center staff should contact the patient who has lost follow-up in order to determine the reason and try to reschedule the visit. The date the patient was contacted, and the contact details should be recorded in the study file).<br><br>5. Evaluated by investigator<br><br>1) If, for any reason, the patient needs to be treated with another drug that has been shown to be effective in treating the indication of the study, the patient should withdraw from the study before using the new drug.<br><br>2) Disease progression or further treatment is deemed unsuitable by the investigator.

	3) Any treatment-related event considered life-threatening has occurred, regardless of the severity of the event.<br>4) Study drugs meet withdrawal criteria due to toxicity.<br>5) Patient or client (such as parent or legal guardian) requests to withdraw from the study or discontinuation of study drugs (if patient withdraws informed consent for treatment but not for follow-up, long-term follow-up is still available.<br>6) The Investigator or co-sponsors may terminate the study or discontinue the patient's participation for medical, safety, regulatory, or other reasons related to GCP.<br>Note: Reasons for termination and termination dates for all patients will be collected.
Drugs,<br>Dose, Route,<br>Regimen	（1）Camrelizumab<br>- Specification: 200 mg/ bottle<br>- Administration method: 200mg IV D1 Q3W<br>（2）Carboplatin<br>- Specification: 150mg/ piece<br>- Administration method: AUC (mg/mL/min) set 5, total carboplatin (mg)= Set AUC× (Ccr +25) IV D1 Q3W<br>（3）Nab-paclitaxel<br>- Specification: 100 mg/ piece<br>- Administration method: 260mg/m^2 IV D1 Q3W

Evaluation Criteria	(1) Safety evaluation:<br><br>The incidence and severity of all Adverse Events (AE), Treatment Emergent Adverse Even (TEAE), Adverse Events of Special Interest (AESI), and Serious Adverse Events (SAE) were evaluated according to CTCAE version 5.0.<br><br>The changes of vital signs, physical examination results, and laboratory results before, during, and after treatment.<br><br>Assessment of surgical safety: surgical R0 resection rate, operation duration, blood loss, chest drainage fluid volume, length of hospital stay, incidence of complications, perioperative mortality, etc.<br><br>(2) Effectiveness evaluation:<br><br>The ORR and DCR were assessed according to RECIST1.1 criteria.<br><br>Pathological evaluation: MPR rate, PCR rate, R0 resection rate, tumor infiltrating lymphocytes (TIL), tumor cell death rate, etc.<br><br>Follow-up of survival: Disease-Free Survival (DFS) and Overall Survival (OS).<br><br>(3) Biomarkers:<br><br>Tumor tissue samples were collected for tumor biomarker analysis, including but not limited to the changes of the following indicators in tumor specimens (PD-L1, TMB, TNB, MSI, dMMR, TIL, DDR pathway, etc.).<br><br>Blood samples were collected for circulating tumor cell (CTC) and circulating tumor DNA (ctDNA) analysis, including but not limited to PD-

	L1 dynamic changes.<br>(4) Evaluation of Quality of life:<br>Changes in quality of life and health status of enrolled patients during treatment were assessed according to EORTC QLQ-C30 and EORTC QLQ-OES18.
Study Duration	The duration of enrollment in this study lasts about 2 years, and patients were followed up for long-term survival after the intervention.
Trial Progress	（1）Patients need to complete relevant examinations and evaluations. If the patients meet all inclusion and exclusion criteria, they will be enrolled and sign the informed consent.<br>（2）Enrolled patients will receive 2 cycles of camrelizumab (200mg IV D1 Q3W) combined with carboplatin (AUC set 5, IV D1 Q3W) and nab-paclitaxel (260mg/m2, IV D1 Q3W, 30 min each infusion), and its safety was evaluated during the treatment.<br>（3）After completing 2 cycles of camrelizumab combined with carboplatin and albumin-paclitaxel, the patient needs to return to the hospital for re-examination of physical examination, blood biochemical examination, liver and kidney function examination. PET/CT or neck, chest and upper abdomen CT scan needs to be performed again. Patients suspected of distant metastasis should be performed examination of the specific site to confirm the diagnosis. At the same time, relevant preoperative examination should be performed to exclude surgical contraindications. Efficacy was evaluated according to RECIST 1.1 criteria.<br>（4）Surgical treatment: the surgical method adopted in this study was

	radical resection of esophageal cancer through three incisions of right chest, neck and upper abdomen (Mckeown). Surgery is performed within 3-6 weeks after completion of the second chemotherapy. The scope of lymph node dissection: lymph nodes in the thoracic and upper abdominal surgical fields. Surgical margin: the proximal and distal margin should be over 5cm beyond the edge of tumor. Pathology was used to determine the depth of tumor invasion, whether the resection margin contained tumor cells, and the proportion of tumor cells. This procedure is used to assess R0 resection, pathological response rate, and subsequent treatment decisions.<br>（5）Camrelizumab maintenance therapy (200mg IV D1 Q3W) was started 4-8 weeks after surgery, and the maintenance therapy lasts a year after surgery. (Note: Maintenance therapy will not be carried out for serious infection or other conditions that are not suitable for maintenance therapy after operation.)<br>（6）After treatment, regular follow-up of survival was performed every 3 months in the first year after surgery, and every 6 months in 2-5 years after surgery, including recurrence and related treatment.
Safety	According to the mechanism of camrelizumab and clinical safety information of products with the same mechanism, adverse events may occur in the process of this clinical trial are primarily reactive capillary hyperplasia and immunity inflammation caused by the immune system activation such as pneumonia, colitis, hepatitis, nephritis, renal insufficiency and inflammation of the endocrine system, etc. According to the clinical data of existing anti-PD-1 monoclonal antibody drugs, although the incidence of adverse events is high, however these are

	tolerated. Only a small part of patients will stop taking drugs due to adverse reactions, and most of the adverse reactions can be relieved after treatment. For early symptoms of immune related adverse reaction are varied, special attention should be paid to early signs and symptoms of various immune related reaction to make correct judgement in time and perform dose adjustment according to the plan and give the specific treatment. Reduce the risk of patients who use the drugs. At the same time, attention should be paid to exclude patients with autoimmune diseases to avoid exacerbation of the original disease caused by activation of the immune system.<br><br>Phase I-III clinical pharmacology and safety data of camrelizumab indicate that camrelizumab has definite pharmacological activity and is well tolerated in patients with advanced tumors.<br><br>The data above preliminarily indicate that SHR-1210 (camrelizumab) has satisfying safety and pharmacological activity. And similar drugs have significant antitumor activity in advanced esophageal cancer patients that supports conducting clinical studies in esophageal cancer patients in China.
Statistical Methodology	The sample size was 20 cases. The detailed statistical method is described in the study protocol.

1.Background and rationale

1.1 Epidemiology of esophageal cancer.

Esophageal cancer (EC) is one of the malignant tumors that seriously threaten human health. The pathology of EC are mainly squamous cell carcinoma and adenocarcinoma. China has a high prevalence of EC and is home to more than half of the EC patients in the world. According to the latest cancer statistics in China, the incidence and mortality of esophageal cancer rank third and fourth among all kinds of malignant tumors[1]. The main pathological type is squamous cell carcinoma in the Asian populations, while adenocarcinoma is the main type of esophageal cancer in western countries. There are many differences between esophageal squamous cell carcinoma (ESCC) and adenocarcinoma carcinoma in pathophysiology and pathogenesis[2]. It is of great significance to study the prevention and treatment of ESCC.

Most EC cases are initially diagnosed at an advanced stage of the disease[3]. In the past 30 years, although emphasis has been placed on multidisciplinary comprehensive treatment of ESCC, the overall survival of ESCC patients has improved little, with a 5-year survival rate of only 15-25%[4]. Due to the limited efficacy of chemotherapy and radiotherapy in the treatment of ESCC, it is easy to recur and metastasize[5].

1.2 Efficacy and deficiency of neoadjuvant chemotherapy in the treatment of stage II-III esophageal cancer.

Surgical treatment is still the main therapy for the treatment of stage II-III esophageal cancer. However, clinical studies have shown that the pathological R0 resection rate of these patients was only 60% and resulted early esophageal recurrence in 58% patients, and the median survival time is only about 17 months[6]. Therefore, for patients with stage II-III esophageal cancer, how to improve the R0 resection rate and reduce postoperative local recurrence is one of the important directions to improve the prognosis of esophageal cancer.

The purpose of neoadjuvant chemotherapy is to increase the R0 resection rate and improve the overall prognosis of patients. Some studies have been carried out in neoadjuvant chemotherapy for esophageal cancer. The early application of cisplatin and fluorouracil improved the curative resection rate. And the neoadjuvant chemotherapy had a better OS (5-year rate 38% vs 24%) and a better disease-free survival (5-year rate: 34% vs 19%)[7]. In recent years, paclitaxel drugs were added, and the efficiency and pathological complete response (pCR) rate were further improved. In a study of 209 patients with stage III esophageal cancer, traditional CF regimen combined with docetaxel (DCF) regimen was used in preoperative neoadjuvant chemotherapy. The results showed that the total effective rate of DCF regimen was significantly better than that of CF regimen[8]. Another domestic study on locally advanced esophageal squamous cell carcinoma showed that in preoperative neoadjuvant chemotherapy for esophageal squamous cell carcinoma, docetaxel combined with cisplatin has better clinical tolerance

than traditional CF regimen, and the postoperative pathological complete remission rate also had a significant advantage[9]. However, the side effects of neoadjuvant chemotherapy such as severe neutropenia, fever, nausea and vomiting could not be ignored[10]. If the neoadjuvant chemotherapy is ineffective, it will undoubtedly increase the financial burden of patients and may lead to the loss of surgical opportunities, and the worse prognosis.

1.3 Neoadjuvant chemotherapy and neoadjuvant chemoradiotherapy.

Neoadjuvant chemoradiotherapy and neoadjuvant chemotherapy are the main neoadjuvant therapy modes for esophageal cancer. NEOCRTEC 5010 and CROSS study both demonstrated that neoadjuvant chemoradiotherapy plus surgery improves survival over surgery alone among patients with locally advanced EC, extending median overall survival by 33.6 months and 60.5 months, respectively[11-12]. Therefore, for patients with resectable esophageal cancer (clinical stage T1B-T2 N + or T3-T4A with any N or suspected involvement of surrounding organs but no T4b is identified), the CSCO Guidelines recommended preoperative neoadjuvant chemoradiotherapy (class 1A evidence) or neoadjuvant chemotherapy (class 1B evidence) [13].

However, due to the toxicity of neoadjuvant chemoradiotherapy, most of the patients are prone to intolerance and suffer poor nutritional status, decreased KPS score and quality of life [14-15]. Moreover, preoperative chemoradiotherapy require close cooperation with multi-disciplinary such as pre-treatment assessment, radiotherapy, surgery, nutrition, perioperative care and pathology. The application of neoadjuvant chemoradiotherapy in clinical practice is limited.

In addition, neoadjuvant chemoradiotherapy has not been able to improve the status of locally advanced esophageal cancer with tumor recurrence. In NEOCRTEC 5010, distant recurrence accounted in 71.0% of all patients with recurrence, and distant recurrence accounted for 80.5% of all patients in CROSS study. It is suggested that the recurrence mode after neoadjuvant chemoradiotherapy is mainly distant metastasis, and there is still no effective systemic treatment for locally advanced esophageal cancer. A more effective and less toxic neoadjuvant treatment regimen is therefore needed to improve the clinical outcomes of ESCC patients without increasing the burden of treatment-related adverse events.

1.4 Immunotherapy for esophageal cancer

PD-1 inhibitors have achieved encouraging antitumor efficacy in a variety of tumors. In recent years, some clinical studies have shown the efficacy of PD-1 inhibitors in advanced esophageal cancer. The KEYNOTE-028 study enrolled 23 PD-L1-positive patients who received pembrolizumab. 29% (5/ 17) ESCC patients and 40% (2/5) esophageal adenocarcinoma achieve partial response (PR) and the overall ORR was 30% [16]. KEYNOTE-180 and KEYNOTE-181 is recruiting patients. A phase II study from Japan reported that the ORR of nivolumab for advanced ESCC was 17%, and the median OS was 23 months [17]. In another phase II clinical study of nivolumab in advanced esophageal cancer in which at least first-line treatment failed or intolerable patients, the ORR was 17.2% and mOS was 12.1

months[18]. The SHR-1210 study demonstrated the safety and efficacy of camrelizumab in the treatment of advanced esophageal cancer. The ORR was 33.3% in 30 enrolled patients with advanced ESCC[19]. The ESCORT study was lunched to evaluate the efficacy of camrelizumab in the treatment of advanced esophageal squamous cell carcinoma and announced that the study has reached the main end point. The results of the study will be published in the near future. At the 2018 CSCO meeting, Professor Shen Lin presented a phase 3 study of a new anti-PD-1 drug tislelizumab in the treatment of advanced esophageal squamous cell carcinoma and reported that tislelizumab achieve an overall ORR of 40%[20]. Based on the above studies, PD-1 blockade induced tumor regression in patients with advanced esophageal squamous cell carcinoma and suggested that immunotherapy would become an effective therapy for the treatment of advanced esophageal cancer in the future.

1.5 Rationale for immunotherapy combined with chemotherapy for resectable esophageal squamous cell carcinoma

Rationale for carboplatin and nab-paclitaxel in the neoadjuvant therapy of esophageal cancer

Carboplatin combined with paclitaxel is also one of the recommended first-line regimens for neoadjuvant therapy in esophageal squamous cell carcinoma in the NCCN guideline[21]. The efficacy of nab-paclitaxel and paclitaxel was comparable and nab-paclitaxel produced less severe adverse events such as neuropathy, neutropenia, myalgia, and arthralgia compared with paclitaxel in the first-line therapy of patients with advanced esophageal cancer and non-small-cell lung cancer [22-23]. In addition, to our knowledge, although there is lack of head-to-head study to compare the efficacy of carboplatin/paclitaxel and cisplatin/FU for neoadjuvant therapy in ESCC, it was reported that the efficacy of carboplatin and paclitaxel (CROSS) was comparable with that of cisplatin and fluorouracil (CALGB 9781) in the neoadjuvant chemoradiotherapy setting (median overall survival: 49.4 months vs 53.76 month)[12,24].

Rationale for neoadjuvant immunotherapy in neoadjuvant chemotherapy for esophageal cancer

According to the existing research results, immunotherapy shows good anti-tumor efficacy in patients with advanced esophageal cancer, which can improve the objective response rate and prolong survival. Moreover, the PD-1 blockade also showed good safety had a manageable treatment-related adverse effects. Can the immunotherapy also benefit patients with potential resectable tumors? In recent years, immunotherapy has made a breakthrough in the neoadjuvant therapy of lung cancer. In a small sample clinical study of nivolumab for neoadjuvant therapy of lung cancer, the pathological remission rate reached 45%, and the side effects were acceptable. Neoadjuvant immunotherapy did not lead to the delay of surgery [25]. The research results of immunotherapy in neoadjuvant therapy of lung cancer suggested that the block of PD-1 pathway in patients with early lung cancer may increase host immune adaptability and reduce tumor heterogeneity, which may enhance the anti-tumor effect. At present, the role of immunotherapy in neoadjuvant therapy for esophageal cancer is not clear. It is of great significance to further clarify the safety and effectiveness of immunotherapy in neoadjuvant therapy for esophageal cancer. It is of great simportance to explore a new treatment model and improve the effective rate of neoadjuvant therapy for esophageal cancer.

Rationale for immunotherapy combined with chemotherapy for resectable esophageal squamous cell carcinoma

Preclinical studies have confirmed that PD-1 inhibitors combined with chemotherapy can further enhance host's immune response and inhibit cancer cell immune escape[26]. Chemotherapy could disrupt the activity of immunosuppressive cells such as regulatory T cells (Treg), myeloid suppressor cells (MDSC), and tumor-associated macrophages (TAM). Moreover, chemotherapy can promote immune response by inducing tumor cell apoptosis, up-regulating of MHC-1 molecule expression and dendritic cell maturation[27].

1.6 References:

1. Chen W. Cancer statistics: updated cancer burden in China. *Chinese journal of cancer research = Chung-kuo yen cheng yen chiu* 2015;27:1.
2. Abnet C, Arnold M, Wei W. Epidemiology of Esophageal Squamous Cell Carcinoma. *Gastroenterology* 2018;154:360-373.
3. Chen W, Zheng R, Baade P, et al. Cancer statistics in China, 2015. *CA: a cancer journal for clinicians* 2016;66:115-132.
4. Pennathur A, Gibson M, Jobe B, et al. Oesophageal carcinoma. *Lancet (London, England)* 2013;381:400-412.
5. Rustgi A, El-Serag H. Esophageal carcinoma. *The New England journal of medicine* 2014;371:2499-2509.
6. Ning Z, Zhao W, Li X, et al. The status of perineural invasion predicts the outcomes of postoperative radiotherapy in locally advanced esophageal squamous cell carcinoma. *International journal of clinical and experimental pathology* 2015;8:6881-6890.
7. Ychou M, Boige V, Pignon J, et al. Perioperative chemotherapy compared with surgery alone for resectable gastroesophageal adenocarcinoma: an FNCLCC and FFCD multicenter phase III trial. *Journal of clinical oncology : official journal of the American Society of Clinical Oncology* 2011;29:1715-1721.
8. Nomura M, Oze I, Abe T, et al. Impact of docetaxel in addition to cisplatin and fluorouracil as neoadjuvant treatment for resectable stage III or T3 esophageal cancer: a propensity score-matched analysis. *Cancer chemotherapy and pharmacology* 2015;76:357-363.
9. Wu S, Chen M, Luo J, et al. [Comparison between docetaxel plus cisplatin and cisplatin plus fluorouracil in the neoadjuvant chemoradiotherapy for local advanced esophageal squamous cell carcinoma]. *Zhonghua zhong liu za zhi [Chinese journal of oncology]* 2012;34:873-876.
10. Ilson D, Forastiere A, Arquette M, et al. A phase II trial of paclitaxel and cisplatin in patients with advanced carcinoma of the esophagus. *Cancer journal (Sudbury, Mass)* 2000;6:316-323.
11. Yang H, Liu H, Chen Y, et al. Neoadjuvant Chemoradiotherapy Followed by Surgery Versus Surgery Alone for Locally Advanced Squamous Cell Carcinoma of the Esophagus (NEOCRTEC5010): A Phase III Multicenter, Randomized, Open-Label Clinical Trial. *Journal of clinical oncology : official journal of the American Society of Clinical Oncology* 2018;36:2796-2803.
12. van Hagen P, Hulshof M, van Lanschot J, et al. Preoperative chemoradiotherapy for esophageal or junctional cancer. *The New England journal of medicine* 2012;366:2074-2084.
13. Guidelines for esophageal cancer. *CSCO: Chinese society of clinical oncology* 2020.

14. Mariette C, Dahan L, Mornex F, et al. Surgery alone versus chemoradiotherapy followed by surgery for stage I and II esophageal cancer: final analysis of randomized controlled phase III trial FFCD 9901. *Journal of clinical oncology : official journal of the American Society of Clinical Oncology* 2014;32:2416-2422.

15. Chan K, Saluja R, Delos Santos K, et al. Neoadjuvant treatments for locally advanced, resectable esophageal cancer: A network meta-analysis. *International journal of cancer* 2018;143:430-437.

16. Doi T, Piha-Paul S, Jalal S, et al. Safety and Antitumor Activity of the Anti-Programmed Death-1 Antibody Pembrolizumab in Patients With Advanced Esophageal Carcinoma. *Journal of clinical oncology : official journal of the American Society of Clinical Oncology* 2018;36:61-67.

17. Kudo T, Hamamoto Y, Kato K, et al. Nivolumab treatment for oesophageal squamous-cell carcinoma: an open-label, multicentre, phase 2 trial. *The Lancet Oncology* 2017;18:631-639.

18. Tsushima HY, Takashi Ura, Kei Muro, Taroh Satoh, Yuichiro Doki, Atsushi Ohtsu, YasuoHamamoto, Yuko Kitagawa. Phase II study of nivolumab (ONO-4538/BMS-936558) in patients with esophageal cancer: Preliminary report of overall survival. *J Clin Oncol* 2016;suppl 4S;abstract 175.

19. Huang J, Xu B, Mo H, et al. Safety, Activity, and Biomarkers of SHR-1210, an Anti-PD-1 Antibody, for Patients with Advanced Esophageal Carcinoma. *Clinical cancer research : an official journal of the American Association for Cancer Research* 2018;24:1296-1304.

20. Lim S, Hong M, Ahn S, et al. Changes in tumour expression of programmed death-ligand 1 after neoadjuvant concurrent chemoradiotherapy in patients with squamous oesophageal cancer. *European journal of cancer (Oxford, England : 1990)* 2016;52:1-9.

21. Esophageal and Esophagogastric Junction Cancers.Version 2.2019. *National Comprehensive Cancer Network* 2019.

22. Socinski M, Bondarenko I, Karaseva N, et al. Weekly nab-paclitaxel in combination with carboplatin versus solvent-based paclitaxel plus carboplatin as first-line therapy in patients with advanced non-small-cell lung cancer: final results of a phase III trial. *Journal of clinical oncology : official journal of the American Society of Clinical Oncology* 2012;30:2055-2062.

23. Wang H, Yao Z, Tang H, et al. Weekly nanoparticle albumin-bound paclitaxel in combination with cisplatin versus weekly solvent-based paclitaxel plus cisplatin as first-line therapy in Chinese patients with advanced esophageal squamous cell carcinoma. *OncoTargets and therapy* 2016;9:5663-5669.

24. Tepper J, Krasna M, Niedzwiecki D, et al. Phase III trial of trimodality therapy with cisplatin, fluorouracil, radiotherapy, and surgery compared with surgery alone for esophageal cancer: CALGB 9781. *Journal of clinical oncology : official journal of the American Society of Clinical Oncology* 2008;26:1086-1092.

25. Forde P, Chaft J, Smith K, et al. Neoadjuvant PD-1 Blockade in Resectable Lung Cancer. *The New England journal of medicine* 2018;378:1976-1986.

26. Emens L, Middleton G. The interplay of immunotherapy and chemotherapy: harnessing potential synergies. *Cancer immunology research* 2015;3:436-443.

27. Mathew M, Enzler T, Shu C, et al. Combining chemotherapy with PD-1 blockade in NSCLC. *Pharmacology & therapeutics* 2018;186:130-137.

2. Objectives

2.1 Primary

To evaluate the safety and feasibility of camrelizumab combined with carboplatin and nab-paclitaxel in the neoadjuvant therapy of potentially resectable stage II-III esophageal squamous cell carcinoma.

2.2 Secondary

1) To evaluate the efficacy of camrelizumab combined with carboplatin and nab-paclitaxel chemotherapy in neoadjuvant therapy for potentially resectable esophageal squamous cell carcinoma in stage II-III (ORR, DCR, MPR, R0 resection rate, etc.)
2) To evaluate the quality of life of patients diagnosed with stage II-III potentially resectable esophageal squamous cell carcinoma who received neoadjuvant therapy with carboplatin and albumin-paclitaxel chemotherapy；
3) To evaluate the disease-free survival (DFS) of camrelizumab combined with carboplatin and nab-paclitaxel in neoadjuvant therapy for stage II-III potentially resectable esophageal squamous cell carcinoma；
4) To evaluate the overall survival (OS) of neoadjuvant therapy with camrelizumab combined with carboplatin and nab-paclitaxel for stage II-III potentially resectable esophageal squamous cell carcinoma;
5) To evaluate the correlation between immune-related markers (PD-L1, TMB, TNB, MSI, dMMR, TIL, DDR pathway, etc.) and the efficacy of neoadjuvant therapy with camrelizumab for stage II-III potentially resectable esophageal squamous cell carcinoma.

Yang W, *et al. J Immunother Cancer* 2022; 10:e003497. doi: 10.1136/jitc-2021-003497

3. Study design

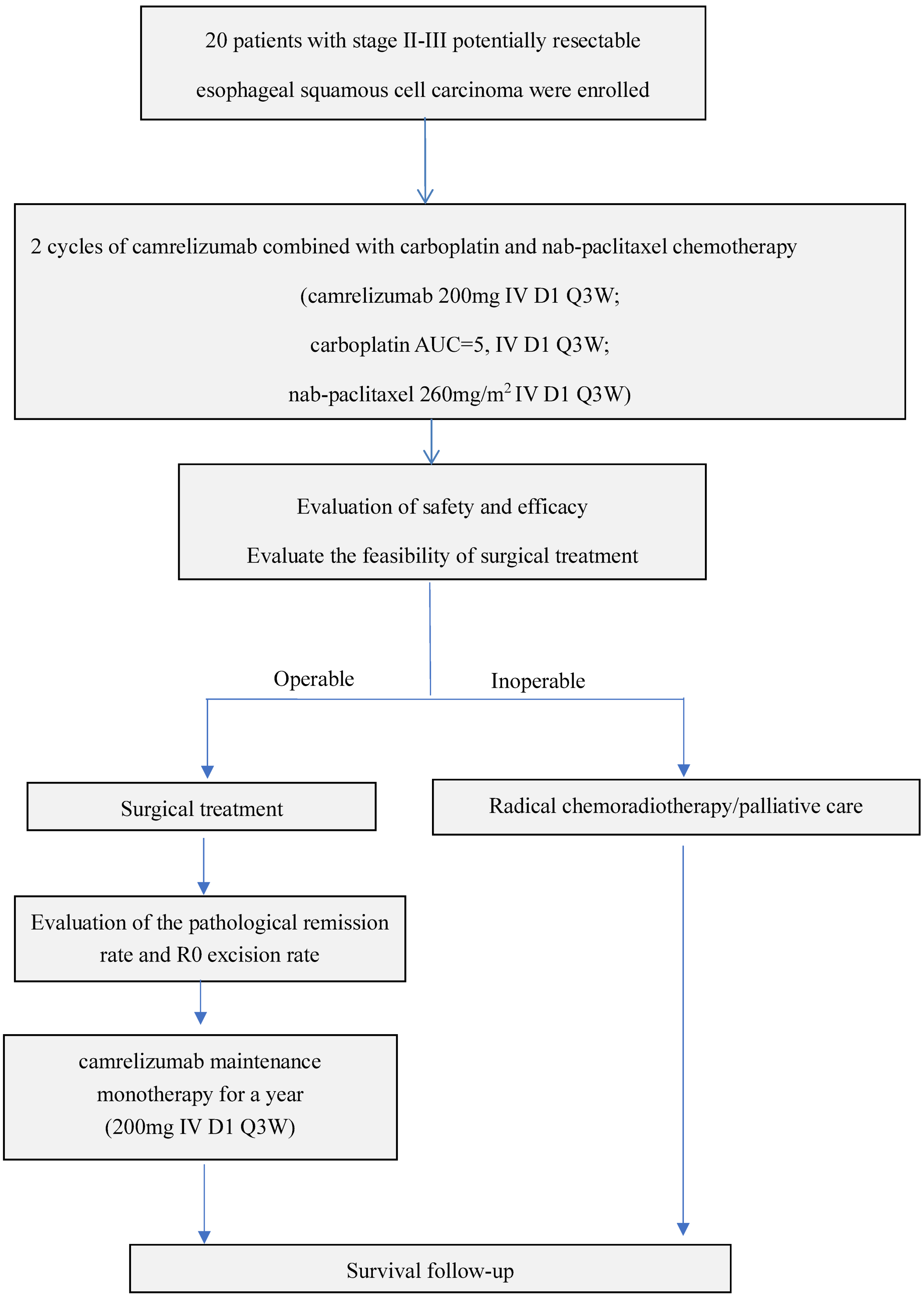

4. Patient selection

4.1 Inclusion Criteria

1) The patient's pathological biopsy results must be confirmed to be esophageal squamous cell carcinoma (not including mixed adenosquamous carcinoma or other pathological types) by pathologists.

2) Age ≥18 y and ≤75 y.

3) ECOG or PS score is 0 or 1.

4) Patients did not previously receive cancer related chemotherapy, radiotherapy, or surgery.

5) Endoscopically diagnosed cervical esophageal cancer and gastroesophageal junction tumors were not included in this study.

6) Clinical stage should be T2-3/N0-2/M0 (II-III), with potential of radical surgical treatment.

7) Patients should have PET-CT examination or neck/chest +upper abdomen CT scan, ECT, brain MR/CT, etc. to clarify staging and exclude distant metastasis.

8) Adequate organ and marrow function as defined below:

i. Blood routine examination: Absolute neutrophil count (ANC) ≥1.5×109/L; Platelet count (PLT) ≥100×109/L; Hemoglobin content (HGB) ≥ 9.0g /dL.

ii. Liver function: serum total bilirubin (TBIL) ≤1.5×Upper Limit Of Normal (ULN); Alanine aminotransferase (ALT) and aspartate aminotransferase (AST) ≤2.5×ULN.

iii. Renal function: Creatinine clearance (Ccr) ≥60 mL/min (calculated by Cockcroft/Gault formula) :

$$female: Ccr = \frac{(140 - age) \times weight(kg) \times 0.85}{72 \times Scr\left(\frac{mg}{dL}\right)}$$

$$male: Ccr = \frac{(140 - age) \times weight(kg) \times 1.00}{72 \times Scr(mg/dL)}$$

iv. Adequate coagulation function, defined as international normalized ratio (INR) or prothrombin time (PT) ≤1.5 ULN; If patient is receiving anticoagulant therapy, it is acceptable that PT is in the prescribed anticoagulant range.

9) Female patients of reproductive age or male patients whose sexual partner is female patients of reproductive age shall use effective contraceptive measures throughout the treatment period until 6 months after the treatment period.
10) Sign written informed consent and be able to comply with the visits and related procedures specified in the program.
11) Can provide archived pathological tissues or fresh pathological tissues within 6 months for the detection of PD-L1 and other markers.

4.2 Exclusion Criteria

1) Patients were taking other investigational drugs at the same time.

2) Those who have not recovered from recent major surgical procedure.

3) Any treatment with anti-PD-1, anti-PD-L1, anti-PD-L2, anti-CD137, or anti-CTLA-4 antibodies, or any other antibody or drug that specifically targets at T-cell costimulation or checkpoint pathways prior to study.

4) History of allergic reactions attributed to any monoclonal antibody or chemotherapy drug (paclitaxel, carboplatin) preparations or excipients.

5) Patients were also taking rifampicin, phenytoin sodium, carbamazepine, or barbiturates (these drugs induce CYP3A and may reduce plasma content of paclitaxel).

6) Received systemic therapy of Chinese herbal medicine with anti-tumor indications or immunomodulatory drugs (including thymosin, interferon, interleukin, etc.) within 2 weeks prior to first administration.

7) Administration of a live, attenuated vaccine within 4 weeks prior to the first dose of treatment or planned for it during the study.

Note: Administration of inactivated virus vaccine for seasonal influenza is permitted within 4 weeks prior to the first dose of treatment， while live attenuated flu vaccines are not allowed.

8) Toxicity from prior antineoplastic therapy that did not return to grade 0 or 1 defined by National Cancer Institute General Adverse Event Terminology version 5.0(NCI CTCAE version 5.0, excluding alopecia, non-clinically significant or asymptomatic laboratory abnormalities).

9) Known autoimmune disease that needs symptomatic treatment or history of disease within 2 years (patients with vitiligo, psoriasis, hair loss, or grave disease that doesn't need systemic treatment, hypothyroidism that only needs thyroid hormone replacement therapy and type 1 diabetes which only need insulin replacement therapy can be enrolled).

10) Known history of primary immunodeficiency.

11) Known to have active infection of tuberculosis.

12) Known history of allogeneic organ transplantation and allogeneic hematopoietic stem cell transplantation.

13) HIV infection and carriers are known to exist (HIV antibody positive).

14) Severe infections that are in active period or clinically poorly controlled.

15) Symptomatic congestive heart failure (NYHA II-IV) or symptomatic or poorly controlled arrhythmia.

16) Uncontrolled arterial hypertension (systolic blood pressure ≥160mmHg or diastolic blood pressure ≥100mmHg) even with standard therapy.

17) Any arterial thromboembolic event, including myocardial infarction, unstable angina, cardiocerebral events or transient ischemic attack (TIA), occurred within 6 months prior to enrollment.

18) Significant malnutrition which need intravenous supplements while malnutrition corrected for over 4 weeks prior to first dose of the study was excluded.

19) A history of deep vein thrombosis, pulmonary embolism, or any other severe thromboembolism within 3 months prior to enrollment (Implantable Venous Access Port or duct-derived thrombosis, or superficial venous thrombosis is not considered as "severe" thromboembolism).

20) Uncontrolled metabolic disorders or other non-malignant organ or systemic diseases or secondary reactions to cancer that may result in higher medical risk and/or uncertainty in the assessment of survival.

21) Hepatic encephalopathy, hepatorenal syndrome，Child-Pugh B liver cirrhosis or worse.

22) History of intestinal obstruction or following diseases: inflammatory bowel disease (IBD) or extensive bowel resection (partial resection of the colon or extensive resection of the small intestine with chronic diarrhea), Crohn's disease, ulcerative colitis (UC).

23) Known to have acute or chronic active hepatitis B (HBsAg positive with HBV DNA viral load ≥ 10^3 IU/ml /mL or >200IU/ mL) or acute or chronic active hepatitis C (HCV antibody positive and HCV RNA positive).

24) History of gastrointestinal perforation and/or fistula within 6 months prior to enrollment.

25) Interstitial lung disease (ILD) requiring steroid therapy.

26) History of other primary malignancies, excluding:

i Complete remission (CR) of malignant tumors for at least 2 years before enrollment and no other treatment was required during the study;

ii Non-melanoma skin cancer or Lentigo maligna (LM) that has been adequately treated and has no evidence of disease recurrence;

iii Adequately treated carcinoma in situ (CIS) with no evidence of disease recurrence.

27) Pregnant or lactating female patients.

28) Other acute or chronic diseases, psychiatric disorders, or abnormal laboratory test values that may cause：increase related risk of study participation or drug administration, interfere with the interpretation

of study results or ineligible patients for study participation from researcher's judgment.

29) Patients with difficulty in obtaining satisfactory biopsy specimens through endoscopy for relevant detection of the study.

4.3 Withdraw criteria

1. The patient is found to be ineligible for the inclusion/exclusion criteria and is deemed unsuitable for further study by the investigator.

2. The patient violates the study protocol, and it is considered inappropriate to continue to participate in further study by the investigator.

3. Any other type of pharmaceutical research that is considered scientifically or medically incompatible with this study.

4. The patient fails to finish the defined follow-up evaluations (Research center staff should contact the patient who has lost follow-up in order to determine the reason and try to reschedule the visit. The date the patient was contacted, and the contact details should be recorded in the study file).

5. Evaluated by investigator

1) If, for any reason, the patient needs to be treated with another drug that has been shown to be effective in treating the indication of the study, the patient should withdraw from the study before using the new drug.

2) Disease progression or further treatment is deemed unsuitable by the investigator.

3) Any treatment-related event considered life-threatening has occurred, regardless of the severity of the event.

4) Study drugs meet withdrawal criteria due to toxicity.

5) Patient or client (such as parent or legal guardian) requests to withdraw from the study or discontinuation of study drugs (if patient withdraws informed consent for treatment but not for follow-

up, long-term follow-up is still available.

6) The Investigator or co-sponsors may terminate the study or discontinue the patient's participation for medical, safety, regulatory, or other reasons related to GCP.

Note: Reasons for termination and termination dates for all patients will be collected.

5. Treatment Plan

5.1 Neoadjuvant administration Regimen

Camrelizumab, 200mg IV D1 Q3W, maintain 30 to 60 minutes per infusion; Carboplatin AUC=5, IV D1 Q3W; Nab-paclitaxel, 260 mg/m2 IV D1Q3W, maintain 30 minutes per infusion. Chemotherapy should be started 1 hour after the end of camrelizumab infusion and finish monitoring of vital signs.

Camrelizumab 200 mg fixed dose (3mg/kg for patients weighing less than 40kg) is administered intravenously every 3 weeks. 200mg of each camrelizumab freeze-dried powder injection was first redissolved with 5mL of water, and then further diluted with 100mL 5% glucose or 0.9% sodium chloride, each infusion maintains 30-60 minutes.

For patients with normal renal function, the recommended dose of carboplatin is calculated by AUC set as 5, and a single dose of carboplatin is given intravenously for over 15-60 minutes every 3 weeks. Neutrophil count ⩾2000/mm^3; Platelet count ⩾100000/mm^3 is required for the next cycle of treatment. During configuration, the product should be dissolved with 5% glucose injection at a concentration of 10mg/ml, and then add it into 250-500ml 5% glucose for injection intravenously.

Nab-paclitaxel 260 mg/m^2 was given intravenously for over 30 minutes and observed for 30 minutes after infusion. The total dose per cycle was 260 mg/m^2.

5.2 Surgical plan and Procedure:

The operation was performed 3-6 weeks after the end of the second neoadjuvant chemotherapy, and the surgical method adopted in this study was radical resection of esophageal cancer through three incisions of right chest, neck and upper abdomen (Mckeown).

5.2.1 Preoperative preparation of patients

1) Psychological nursing: communication and reduce the apprehension.
2) Nutritional support: food with high nutrition, rich in protein and vitamin.
3) Preparation of respiratory tract: quit smoking, effective cough training.
4) Preparation of gastrointestinal tract: fasting for 8 hours and no drinking for 6 hours before surgery.
5) To control comorbidity: hypertension, coronary heart disease, diabetes, nutritional metabolism disorders, etc.

5.2.2 Surgical procedure

Consciousness, body temperature (T), electrocardiogram (ECG), heart rate (HR), blood pressure

(Bp), oxygen saturation (SpO2), respiratory rate (RR) were continuously monitored once patients get into the operating room. After the artificial airway was established, pressure of end-tidal CO2 ($P_{et}CO2$) and fraction of inspiration O_2 (FiO2) were monitored.

Thoracic surgery: thoracoscopic esophageal dissociation and lymph node dissection were performed on the chest, requiring complete dissection of lymph nodes in each regions including para-esophageal lymph nodes, para-laryngeal recurrent nerve lymph nodes and subcarinal lymph nodes. If complete resection could not be performed, metal clips should be used and fill in the form with explanation. Intraoperative transfer to open surgery should be recorded.

Abdominal and neck surgery: abdominal gastric dissociation and abdominal lymph node dissection were performed. Lymph nodes in each region were required to be completely cleaned. If complete resection could not be performed, metal clips were added and instructions were filled in. After the completion of dissociation, the tube stomach can be made through a small open incision in the middle of the abdomen, and the intraoperative transfer should be recorded. In principle, the esophageal bed route is preferred for the gastric ascending route. Special cases can be adjusted according to the experience of the center, which needs to be explained in the form. For neck anastomosis, stapler mechanical anastomosis or manual anastomosis can be selected. No special requirements are required and records should be made. It is necessary to indwelling nasoenteral nutrition tube or jejunal fistula during operation. Whether to indwelling neck drainage tube and gastric tube during the operation according to experience, no special requirements, need to record.

Surgical margin: the proximal and distal resection should be more than 5cm beyond the tumor margin.

5.2.3 Surgical indicators

1) Operation time: total operation duration, chest operation duration, abdominal + neck operation duration.

2) Intraoperative blood loss (ml).

3) Biological specimens will be saved for use, the time and number were recorded.

4) Pathological data, number of stations of dissected lymph nodes and positive number.

5.2.4 Postoperative evaluation

Postoperative adverse reactions and adverse events were recorded and evaluated by the investigator. The records content include:

1) Postoperative weight monitoring, postoperative blood loss, chest tube retain time, drainage flow and color.

2) Occurrence of postoperative complications, including total complications, respiratory complications, cardiovascular complications, recurrent laryngeal nerve injury, chylous leakage, anastomotic fistula, 30-day mortality, etc.

3) Postoperative hospital stay (from end of surgery to discharge), time of postoperative oral feeding, postoperative stitches removal time, duration of ICU stay, and re-admission to ICU.

5.2.5 Discharge and readmission

The patients were evaluated for discharge after reaching the standard. The duration of reaching the standard of discharge after the operation was recorded. Discharge criteria are as follows:

1) Axillary body temperature below 38℃;

2) Blood oxygen saturation >90% without oxygen inhalation,
3) No complications that need hospitalization,
4) For other cases, the investigator will evaluate whether the patient can be discharged.

6. Dose adjustment

6.1 Dose modification of camrelizumab

Table1. Dose Modification Guidelines for Camrelizumab

ADR	Description	Management
Pneumonia	Grade 2	Discontinue for short
	Grade 3 or 4	Discontinue Permanently
Diarrhea/enterocolitis	Grade 2 or 3	Discontinue for short [a]
	Grade 4	Discontinue Permanently
Dermatitis	Grade 3	Discontinue for short [a]
	Grade 4	Discontinue Permanently
Hepatitis	For patients with normal baseline ALT, AST, or TBIL, there appears Grade 2 elevation of AST, ALT, or TBIL. For patients with baseline AST, ALT, or TBIL > ULN, AST, ALT, or TBIL elevates ⩾50% and maintains < 7 days	Discontinue for short [a]
	For patients with normal baseline ALT, AST, or TBIL, there appears Grade 3 or 4 elevation of AST, ALT, or TBIL. For patients with baseline AST, ALT, or TBIL > ULN, AST, ALT, or TBIL elevates ⩾ 50% and maintains ⩾7 days	Discontinue Permanently
Hypophysitis	Grade 2	Discontinue for short [b]
	Grade 3 or 4	Discontinue Permanently
Adrenocortical insufficiency	Grade 2	Discontinue for short [b]
	Grade 3 or 4	Discontinue

		Permanently
Hyperthyroidism	Grade 3 or 4	Discontinue Permanently
Type 1 diabetes	Grade 3	Discontinue for short [b]
	Grade 4	Discontinue Permanently
Renal insufficiency	Grade 2 or 3 / elevation of Cr	Discontinue for short [a]
	Grade 4 / elevation of Cr	Discontinue Permanently
Neurotoxicity	Grade 2	Discontinue for short [a]
	Grade 3 or 4	Discontinue Permanently
Infusion reaction	Grade 3 or 4	Discontinue Permanently
Other AE	Other Grade 3 AE appears for the first time	Discontinue for short [a]
	The same grade 3 AE occurred for second time	Discontinue Permanently
	Grade 3 AE which cannot fall to level 0-2 / baseline within 7 days or recover to level 0-1 / baseline level within 14 days	Discontinue Permanently
	Grade 4 AE	Discontinue Permanently [c]

Note:
a: Resuming dosing after symptom improvement to level 0-1 or baseline.
b: Pituitaritis, adrenocortical insufficiency, and type 1 diabetes mellitus can be re-administered if they are adequately controlled and only physiologic hormone replacement therapy is required.
c: In the case of abnormal grade 4 laboratory results, the decision to discontinue medication should be based on concomitant clinical symptoms/signs and the investigator's clinical judgment.

Table 2. Suggestions for the treatment of Camrelizumab infusion reaction

CTCAE Grade	Dose Modification	Management
Any Grade		- Manage according to local clinical practice<br>- Monitor patients' infusion related reactions (fever or chills, flushing and/or itching, changes in heart rate and blood pressure, dyspnea, chest discomfort, rash,

<table>
<tr><td></td><td></td><td>etc.) and allergic reactions (systemic urticaria, angioedema, asthma, hypotension, tachycardia, etc.)</td></tr>
<tr><td>Grade 1</td><td>The infusion speed can be lowered by 50% or temporarily interrupted until the infusion reaction is relieved</td><td rowspan="2">For Grade 1 or 2 AE:
- Acetaminophen and/or antihistamines will be administered at the investigator's discretion based on local clinical practice
- Consider prophylactic administration prior to subsequent treatment according to local clinical practice</td></tr>
<tr><td>Grade 2</td><td>The infusion speed can be lowered by 50% or temporarily interrupted until the infusion reaction is relieved. The infusion speed can be adjusted to 50% of the initial speed later</td></tr>
<tr><td>Grade 3 or 4</td><td>Discontinue Permanently</td><td>For Grade 3 or 4:
- Manage severe infusion related reactions according to local clinical practice (epinephrine, diphenhydramine, ranitidine, and glucocorticoids)</td></tr>
</table>

6.2 Dose modification of nab-paclitaxel

If the patient's peripheral blood neutrophil count is lower than 1500/mm^3 before treatment, this drug should not be given.

Dose reduction: patients with severe neutropenia (ANC<500/mm^3 for 1 week or more) or severe sensory neurotoxicity during treatment should be treated with a dose reduction to 220mg/m^2 for subsequent courses. In the event mentioned above appears again, the subsequent treatment dose should be reduced to 180mg/m^2. For patients with Grade 3 sensory neurotoxicity, drug administration should be suspended, and the treatment can continue until the neurotoxicity recovered to ⩽ Grade 2, and the dose should be reduced in subsequent treatment.

Table 3. Initial doses are recommended for patients with abnormal liver function

level of AST(SGOT)		Level of Bilirubin	dose of Nab-paclitaxel
≤10×ULN		＞ULN to ≤1.5×ULN	260 mg/m^2
≤10×ULN	And	＞1.5×ULN to ≤3×ULN	220 mg/m^2
≤10×ULN		＞3×ULN to ≤5×ULN	180 mg/m^2
＞10×ULN	Or	＞5×ULN	not recommended

Note: The recommended dose is for the first course only. Dose adjustment requirements for subsequent courses should refer to individual tolerance.

If a patient can tolerate two courses of lower doses, an increased dose to 260 mg/m^2 may be considered in subsequent courses.

Yang W, *et al. J Immunother Cancer* 2022; 10:e003497. doi: 10.1136/jitc-2021-003497

6.3 Dose modification of carboplatin

This product is for intravenous only in patients with normal renal function. According to Calvert formula in Martindale 35th edition: carboplatin dose (mg) = set AUC (mg/ mL /min) × [Ccr (mL /min) +25], the recommended dose is calculated according to AUC=5. Single dose intravenous infusion for over 15-60 minutes. 4 weeks between treatments and/or neutrophil count ≥2000/mm^3; Platelet count ≥100000/mm^3 is required for the next course of treatment. Patients with renal insufficiency had an increased risk of severe myelosuppression when Ccr was less than 60ml/min. The initial dose of carboplatin was adjusted according to Ccr (see Table 4), and the subsequent dose was adjusted according to patient tolerance and acceptable degree of bone marrow suppression.

Table 4. Recommended initial dose for patients with renal dysfunction

Baseline of CCr	Carboplatin initial Dose (Day 1)
41-59 ml/min	250mg/m^2 iv
16-40 ml/min	Stop the medication, blood test once a week, continue the medication after CCr becomes normal

Detection of CCr is complex and is not frequently tested, but Ccr can be calculated through Scr.

Male：CCr (mL /min) ={[140- age (year)]× weight (Kg) ×1.23}÷ SCr (μmol/ L);

Female：CCr (mL /min) = male (mL /min) ×0.85.

For patients with risk factors, such as a history of myelosuppression and poor general condition (ECOG 2-4), a reduction of 20% to 25% of the initial dose is recommended. Initial and subsequent doses should be adjusted for patients aged over 65 years according to their physical status. It is recommended that peripheral blood cell counts should be measured weekly during the first cycle of medication to determine the lowest point of cytopenia in order to adjust the dose for the next cycle.

Yang W, *et al. J Immunother Cancer* 2022; 10:e003497. doi: 10.1136/jitc-2021-003497

Table 5. Dose modification for myelosuppression

WBC	PLT	Dose of Carboplatin
$\geq 4\times10^9$/L	120×10^9/L	100% of the recommended dose
(2.5-3.9) $\times10^9$/L	(75-119) $\times10^9$/L	50% of the recommended dose
$<2.5\times10^9$/L	$<75\times10^9$/L	Stop the medication, blood test once a week, continue the medication after it becomes normal

7. Concomitant medication

7.1 Drugs cannot be used during treatment

1) Other biotherapies (including but not limited to interferon, IL-2, thymosin, immunocell therapy, etc.) and other systemic chemotherapy are prohibited during treatment.
2) Immunotherapy not specified in this protocol is prohibited during treatment.
3) Live vaccine inoculation is prohibited within 28 days before and during drug administration.

7.2 Concomitant therapy during treatment

The conditions permit the use of concomitant drugs during the study are as follows:
1) When adverse reactions occur in the test, it should be strictly observed and treated. All concomitant drugs should be recorded with explanation on the CRF form.
2) When patients vomit due to chemotherapy, antiemetic agents can be given.
3) Neurotrophic agents, such as adenosine cobalamin and vitamin B, can be used when patients develop neurotoxicity.
4) When the patient has pain that affects quality of life, effective analgesic treatment should be given.
5) When the patient has constipation, diarrhea and other symptoms, symptomatic drugs can be given.
6) If severe myelosuppressive toxicity (Grade 3 or 4 toxicity) occurred during the medication, Granulocyte Colony-Stimulating Factor (G-CSF) and other treatment could be given.
7) Bisphosphonates should be used in patients with bone metastasis.
8) Calcium supplements are allowed for treatment.

8.Study process

8.1 Patients need to complete relevant examinations and evaluations. If the patient meets all inclusion

and exclusion criteria, they will be enrolled and sign the informed consent.

(1) Collection of demographic characteristic information.

(2) Information collection of present history, past history and physical examination. Present history and physical examination information should be collected within 28 days of the initial visit.

(4) Height, weight and score of ECOG status.

(5) Complete laboratory tests. All laboratory tests, including routine blood tests, blood biochemistry tests, liver and kidney function tests, and potential infection screening tests, should be completed within 14 days of the first chemotherapy to exclude contraindications related to chemotherapy and immunotherapy.

(6) Pre-treatment examination: Within 28 days of the initial treatment, patients should receive PET-CT examination or enhanced CT of neck, chest and upper abdomen, ECT, and brain MRI for accurate staging and set the baseline of treatment. This is part of standardized treatment. Before treatment, endoscopic ultrasound should be performed to evaluate the depth of invasion, and biopsy should be taken to confirm the pathological diagnosis. At the same time, peripheral blood, biopsy tissue, urine and feces specimens were collected for subsequent detection of immune therapy efficacy indicators. For women of childbearing age, β-HCG test should be performed to exclude pregnancy or early pregnancy.

8.2 Enrolled patients will receive 2 cycles of camrelizumab (200mg IV D1 Q3W) combined with carboplatin (AUC=5 IV D1 Q3W) and nab-paclitaxel (260mg/m2 IV D1 Q3W), and its safety was evaluated during the treatment.

8.3 After completing 2 cycles of camrelizumab combined with carboplatin and albumin-paclitaxel, the patient needs to return to the hospital for re-examination of physical examination, blood biochemical examination, liver and kidney function examination. PET/CT or neck, chest and upper abdomen CT scan needs to be performed again. Patients suspected of distant metastasis should be performed examination of the specific site to confirm the diagnosis. At the same time, relevant preoperative examination should be performed to exclude surgical contraindications. Efficacy was evaluated according to RECIST 1.1 criteria.

8.4 Surgical treatment: the surgical method adopted in this study was radical resection of esophageal cancer through three incisions of right chest, neck and upper abdomen (Mckeown). Surgery is performed within 3-6 weeks after completion of the second chemotherapy. The scope of lymph node dissection: lymph nodes in the thoracic and upper abdominal surgical fields. Surgical margin: the proximal and distal margin should be over 5cm beyond the edge of tumor. Pathology was used to determine the depth of tumor invasion, whether the resection margin contained tumor cells, and the proportion of tumor cells. This procedure is used to assess R0 resection, pathological response rate, and subsequent treatment decisions.

8.5 Camrelizumab maintenance therapy (200mg IV D1 Q3W) was started 4-8 weeks after surgery, and the maintenance therapy lasts a year after surgery. (Note: Maintenance therapy will not be carried out for serious infection or other conditions that are not suitable for maintenance therapy after operation.)

8.6 After treatment, regular follow-up of survival was performed every 3 months in the first year after surgery, and every 6 months in 2-5 years after surgery, including recurrence, survival and related treatment.

9. Adverse events

An adverse event refers to the occurrence or worsening of any clinical symptom, syndrome or disease that occurs during a clinical study and affects the health of the patient. Adverse events may be: new disease; worsening of symptoms or signs or worsening of concomitant disease; the influence of test methods or drugs; A combination of one or more factors.

Any adverse medical event that occurred between the time the patient signed the informed consent and was enrolled in the study and the last visit was considered an adverse event.

9.1 Adverse events include but are not limited to:

(1) All adverse drug reactions.

(2) obviously unrelated diseases, including new diseases and exacerbations of pre-existing diseases.

(3) Injuries and accidents.

9.2 Criteria for the severity of adverse events

(1) Mild: tolerable to the patient, does not affect treatment or follow-up, does not require specific treatment, and has no impact on the rehabilitation of the patient.
(2) Moderate: unbearable to the patient, requiring specific treatment, which has a direct impact on the rehabilitation of the patient.
(3) Severe: life threatening, causing death or disability and requiring emergency treatment.

9.3 Recording and management of adverse events

(1) Record: If serious adverse events (SAE)occur during the trial, the investigator must report to the department responsible for clinical research and the ethics committee within 24 hours or no later than the second working day. The researcher must sign the signature and the date. When, how and to whom a serious adverse event was reported should be recorded in the original data. The main research institutes shall immediately notify all participating hospitals and ensure that all reporting procedures required by laws and regulations are implemented.
(3) Patient management: When adverse events are found, researchers can take necessary treatment according to the condition. All adverse events should be tracked and investigated, and the treatment process and results should be recorded in detail until they are properly resolved or the condition is stable. If the laboratory examination is abnormal, it should be tracked until returning to normal. Follow-up can be conducted in hospital, out-patient department, home visit, telephone, and other forms according to the severity of adverse events. Serious adverse events (including abnormal laboratory tests) that were not resolved at the end of the study or the time the patient dropped out early must be followed up to any of the following conditions:1) event resolved 2) event stabilized 3) event return to baseline 4) it has been determined that the study treatment or participation is not the cause 5) When additional information is not available (patient refuses to provide additional information or remains lost to follow-up)

Some events requiring hospitalization or prolonged hospitalization may not be considered as

serious adverse events, including hospitalization for reasons other than adverse event, and hospitalization for surgical or other purposes scheduled prior to the study.

9.4 Serious adverse events

A serious adverse event is an unexpected medical event occurring during the study period that results in death, life-threatening, hospitalization or prolonged hospitalization, persistent or severe disability, congenital abnormalities/defects and other serious events.

After entering the study, if the patient has serious adverse events, in addition to treatment or rescue, the patient should inform the leader of clinical study, clinical supervisor and ethics committee by telephone/fax within 24 hours after being informed. For all serious adverse events, the investigator should immediately take adequate measure and draft a detailed report of the serious adverse event to be submitted to the relevant administrative authorities and the ethics committee. In case of death related to treatment, the clinical trial of this group should be stopped immediately and report to the ethics committee of the clinical research institution as soon as possible. Keep detailed records and proper storage of relevant information.

9.5 Safety Evaluation

It is mainly the observation and evaluation of adverse events during the study, and recorded in detail in the CRF in time.

9.6 Management of tumor recurrence and metastasis

Patients with tumor recurrence and metastasis during follow-up will be recorded in detail in the CRF and treated according to the current clinical pathway.

Yang W, *et al. J Immunother Cancer* 2022; 10:e003497. doi: 10.1136/jitc-2021-003497

10.Others

10.1 Case Report

During the study, all patients were required to fill in the case report form according to the study schedule and requirements.

10.2 Ethical Requirements

Before the initiation of clinical trial, the protocol shall be signed by the investigator, and the trial protocol shall be reviewed and approved by the ethics committee before it can be implemented. During the period of the trial, if problems occur in the actual implementation of the clinical trial and the plan needs to be revised, the revised trial protocol shall be approved by the ethics committee again before it can be implemented. Any serious adverse events and deaths that occur during the trial should be reported by the investigator to the ethics committee.

The ethics committee should be informed at the end of the study.

10.3 Quality control

Investigators should adopt standard operating procedures to ensure the quality control of clinical trials and the implementation of quality assurance systems. All observations and findings in clinical trials should be verified to ensure the reliability of data and to ensure that conclusions in clinical trials are derived from original data. Quality control must be applied at every stage of data processing to ensure that all data are reliable and processed correctly.

10.4 Training of researchers

Prior to the initiation of clinical trials, the investigator shall be trained by the trial protocol so that the investigator can understand and be familiar with the nature, procedure, function and safety of the trial.

10.5 Improve the compliance of patients

1) The researcher should carefully implement informed consent so that the patients can fully understand the requirements and cooperate with the investigator.

2) Follow up regularly to monitor the compliance of patients. Follow-up should be strengthened for those with poor compliance.

10.6 Storage and summary of data

10.6.1 Data Preservation

All data were stored for five years after the termination of the clinical trial.

10.6.2 Confidentiality

All information related to this study (including but not limited to the following documents: study protocol, Investigator's Brochure and summary report) must be kept strictly confidential. Information related to the study or conclusions drawn from the study may be published by the investigator only with the written consent of the project leader. The researcher should send the paper, abstract, or poster intended for publication or academic lecture to the project leader, who will give a reply within 1 month.

Yang W, *et al. J Immunother Cancer* 2022; 10:e003497. doi: 10.1136/jitc-2021-003497

11. Attachments

Annex 1: TNM staging Criteria for Esophageal cancer (AJCC 8th Edition)

Annex 2: ECOG PS Scoring Criteria

Annex 3: Response Evaluation Criteria in Solid Tumors-version 1.1 (RECIST V1.1)

Annex 4: Evaluation criteria for Common Terminology Criteria for Adverse Events 5.0 (CTCAE V 5.0)

Annex 5: NCCN Guidelines for the Management of Immunotherapy-associated Toxicities (2019 V1.0)

Annex 6: List of pre-inclusion autoimmune diseases

Annex 7: Guidelines for dose adjustment and toxicity management of camrelizumab

Annex 8: Quality of Life Scale of EORTC QLQ-C30 and EORTC QLQ-OES18

Signature of principal investigator:

Date:

Yang W, *et al. J Immunother Cancer* 2022; 10:e003497. doi: 10.1136/jitc-2021-003497
