# Supplementary material for: Neoadjuvant programmed cell death 1 blockade combined with chemotherapy for resectable esophageal squamous cell carcinoma
Source: J Immunother Cancer. 2022 Jan 12;10(1):e003497. doi: 10.1136/jitc-2021-003497 (PMC8756283; doi:10.1136/jitc-2021-003497)
Supplement: Supplementary data [file jitc-2021-003497supp001.pdf]

SUPPLEMENTARY MATERIALS

1. SUPPLEMENTARY METHODS

1.1 Quality of life

Health-related quality of life was assessed using the European Organization for Research and Treatment of Cancer (EORTC) Quality of Life Questionnaire-Core 30 (QLQ-C30) and EORTC Quality of Life Questionnaire-Esophageal Cancer Module-18 (QLQ-OES18) scales[1 2], at the start of neoadjuvant therapy and again before the operation. The EORTC QLQ-C30 scale comprises 30 items that are combined to form 5 functioning scales (physical, role, cognitive, emotional, and social), 3 symptom scales (fatigue, pain, and nausea or vomiting), a global health status quality-of-life scale, and 6 single-item scales (dyspnea, insomnia, appetite loss, constipation, diarrhea, and financial problems). The EORTC QLQ-OES18 scale contains 18 items for patients with esophageal cancer.

1.2 Follow up

The patients were scheduled to commence adjuvant treatment with camrelizumab 4–8 weeks following surgery, once they had made a full recovery. Camrelizumab at a dose of 200 mg was given as adjuvant treatment intravenously every 3 weeks for one year after the operation. Restaging and surveillance CT imaging were performed every 3 months during the 1st year of follow-up, every 6 months during the 2nd and 3rd years, and every year thereafter. The endoscopies would be performed when clinically indicated development of dysphagia or worrisome findings on surveillance CT.

1.3 Immunohistochemical and multiplex immunofluorescence staining

1.3.1 PD-L1 immunohistochemistry

A commercially available PD-L1 immunohistochemistry assay (clone 22C3; DAKO Autostainer Link48; RTU) was used to assess the PD-L1 combined positive score in formalin-fixed tumor diagnostic samples in line with the manufacturer's instructions and international guidelines[3 4]. PD-L1 CPS was assessed in 19/23 patients (82.6%). Samples were considered to be PD-L1-positive if CPS$\geqslant$1 of tumor cells showed membranous PD-L1 expression. When multiple pre-treatment specimens were available for PD-L1 testing, the patient was considered PD-L1-positive if any of the pre-treatment specimens were positive, and the highest percentage of PD-L1 positive tumor cells is reported here[5].

Yang W, et al. J Immunother Cancer 2022; 10:e003497. doi: 10.1136/jitc-2021-003497

1.3.2 CD4, CD8, CD56, CD163, PD-1, GRB, and TIA-1 immunohistochemistry

Selected specimens were assessed by immunohistochemistry as previously described, with minor modifications[6]. Tumor tissues were stained for CD4, CD8, CD56, CD163, PD-1, GRB, and TIA-1, as outlined in the table below.

Antibody	Clone/Company	Dilution
CD4	clone 4B12/ Leica	RTU
CD8	clone 4B11/ Leica	RTU
CD56	clone CD564/ Leica	RTU
CD163	clone 10D6/ Leica	RTU
PD-1	clone UMAB199/ ZSGB-Bio	RTU
GRB	clone 11F1/ Leica	RTU
TIA-1	clone 2G9A10F5/ Gene tech	RTU

1.3.3 Multiplex Immunofluorescence Staining

36 samples were adequate to perform the multiplex immunofluorescence staining from 18 patients. Paired samples (pre and post-treatment) were available for the 18 patients (5 PCR patients and 13 Non-PCR patients). The 4μm-thick slides cut from the Formalin-fixed paraffin embedded (FFPE) blocks were dewaxed in xylene, rehydrated through a decreasing ethanol series and fixed in NBF (10% neutral buffered formalin) for 10 min. Slides were stained to enable the simultaneous visualization of six markers: Abs anti-CD8 (Cat# ab93278, Abcam), anti-PD-1 (Cat# 84651S, CST), anti-PD-L1 (Cat# 13684S, CST), anti-CD163 (Cat# ab182422, Abcam), anti-CK (Cat# Kit-0009, MXB Biotechnologies), on the same slide using PANO 7-plex IHC kit, cat 0004100100 (Panovue, Beijing, China). At the beginning of each staining cycle, microwave-heated treatment in EDTA solution was applied to perform antigen retrieval. After blocking proteins for 10 minutes, these five primary antibodies were sequentially incubated for 30, 30, 30, 60, 60 minutes at 37℃, respectively. Then the incubation of HRP-conjugated secondary antibody and tyramide signal amplification (TSA) with Opal was

followed. Five staining cycles were performed for the following antibodies/fluorescent dyes combinations: anti-CD8/Opal-690, anti-PD-1/Opal-620, anti-PD-L1/Opal-520, anti-CD163/Opal-570, anti-CK/Opal-650. Microwave treatment was performed at each cycle of staining to remove the Ab TSA complex. Finally, all slides were stained with 4'-6'-diamidino-2-phenylindole (DAPI, SIGMA-ALDRICH) for 8 min and enclosed with Mounting Medium 0022001010 (Panovue, Beijing, China).

1.3.4 Multispectral Imaging Analysis

The stained slides were scanned using the TissueFAXS platform (TissueGnostics, Vienna, Austria) at 20× magnification, which captures the fluorescent spectra at 20-nm wavelength intervals from 420 to 720 nm with identical exposure time; and the scans were combined to build a single stack image. Spectral libraries were established from the extracted images in which images of unstained and single-stained slides were applied to extract the spectrum of autofluorescence of tissues and each fluorescein, respectively. The library was then used to unmix the multispectral images (seven colors staining) with the StrataQuest software (TissueGnostics, Vienna, Austria). Using this spectral library, reconstructed images of slides with the autofluorescence removed were acquired for imaging analysis. For each primary antibody, the cut-off value for positivity was determined according to the staining pattern and intensities of all images.

1.4 Next-generation sequencing

1.4.1 DNA Extraction and Quantification

Tumor tissue DNA (tDNA) and whole blood control samples were extracted using the QIAamp DNA FFPE Tissue kit and DNeasy Blood and tissue kit (Qiagen, USA), respectively. Purified DNA was qualified by Nanodrop2000 (Thermo Fisher Scientific, Waltham, MA) and quantified by Qubit 2.0 using a dsDNA HS Assay Kit (Life Technologies, Waltham, MA).

1.4.2 Library Preparation

Yang W, *et al. J Immunother Cancer* 2022; 10:e003497. doi: 10.1136/jitc-2021-003497

Sequencing libraries were prepared using the KAPA Hyper Prep kit (KAPA Biosystems, Wilmington, MA) with an optimized manufacturer's protocol [7]. Briefly, ~1 μg of fragmented genomic DNA was subjected to end-repairing, A-tailing, and ligation with indexed adapters sequentially; these steps were followed by size selection using Agencourt AMPure XP beads (Beckman Coulter, Mississauga, Canada) and PCR amplification using the KAPA Hyper DNA Library Prep Kit (KAPA Biosystems, Wilmington, MA).

1.4.3 Hybridization Capture and Sequencing

A customized next-generation sequencing panel targeting exons of 425 cancer-relevant genes (exons and selected introns) was used for hybridization enrichment. Briefly, indexed DNA libraries were pooled together to a total amount of 2 μg and subjected to probe-based hybridization using IDT xGen Lockdown reagents (Integrated DNA Technologies, Coralville, IA) and Dynabeads M-270 (Thermo Fisher Scientific, Waltham, MA) with an optimized manufacturer's protocol [7]. Libraries captured on-beads were amplified with Illumina p5 and p7 primers in KAPA HiFi HotStart ReadyMix (KAPA Biosystems, Wilmington, MA). The final library was quantified using the KAPA Library Quantification kit (KAPA Biosystems, Wilmington, MA) per the manufacturer's instructions. The Bioanalyzer 2100 (Agilent, Santa Clara, CA) was used to determine the fragment size distribution of the final library. The target-enriched library was then sequenced on HiSeq4000 NGS platforms (Illumina) according to the manufacturer's instructions.

1.4.4 Sequencing Data Processing

Sequencing was performed on the Illumina HiSeq4000 platform followed by data analysis as previously described [7]. The medium depth of coverage after the removal of PCR duplicates is 1000X for tumor tissue specimen and 100X for the whole blood control samples, respectively. Specifically, sequencing data were analyzed by Trimmomatic [8] to remove low-quality (quality <15) or N bases, and then mapped to the human reference genome hg19 using the Burrows-Wheeler Aligner (https://github.com/lh3/bwa/tree/master/bwakit). PCR duplicates were removed with Picard (available at: https://broadinstitute.github.io/picard/). The Genome Analysis Toolkit (GATK) (https://software.broadinstitute.org/gatk/) was used to perform local realignment around indels and base quality reassurance. SNPs and indels were analyzed using VarScan2[9] and the Haplotype Caller/Unified Genotyper in GATK, with the mutant allele frequency (MAF) cutoff as 0.5% for tissue samples, and a minimum of 3 unique mutant reads. Common SNPs were excluded if they had a population frequency of >1% in the 1000 Genomes Project or the Exome Aggregation Consortium

(ExAC) 65000 exomes database. The resulting mutation list was further filtered using an in-house list of recurrent artifacts based on a normal pool of whole blood samples.

Tumor mutational burden was defined as the number of coding somatic base substitutions, and short insertions and deletions (indels) per megabase of genome. Copy number variations were detected using ADTEx (http://adtex.sourceforge.net) with default parameters. Identified genetic alterations, including missense, nonsense, indel splicing, and fusion, were also grouped into different Kyoto Encyclopedia of Genes and Genomes pathways, and we focused on immune-related pathways. Comparisons of proportion between groups were done using Fisher's exact test. A 2-sided P value of <0.05 was considered statistically significant for all tests unless indicated otherwise. All statistical analyses and oncoprint were done in R (v.3.5.3).

2. SUPPLEMENTARY TABLES

Supplemental table S1. Clinical response in the population assessed by RECIST 1.1

Clinical response	Total (n=21)
Complete response	1 (4.8%)
Partial response	18 (85.7%)
Stable disease	2 (9.5%)
Progressive disease	0 (0%)

Supplemental table S2. Clinical response versus pathologic response in the 20 patients who underwent surgery

		Pathologic response		
		PCR (n=5)	MPR (n=10)	No PCR or MPR (n=5)
**Clinical response**	Complete response (n=1)	1 (100.0%)	0 (0.0%)	0 (0.0%)
	Partial response (n=17)	4 (23.5%)	10 (58.8%)	3 (17.7%)
	Stable disease (n=2)	0 (0.0%)	0 (0.0%)	2 (100.0%)

Yang W, *et al. J Immunother Cancer* 2022; 10:e003497. doi: 10.1136/jitc-2021-003497

Supplemental table S3. Pathologic downstaging and recurrence status in 20 patients who underwent tumor resection.

Patient ID	cTNM			Stage	Clinical response	ypTNM			Stage	Pathologic response of primary tumor	Downstaging	Recurrence	Recurrence site
01	T2	N1	M0	II	PR	T0	N0	M0	I	PCR	Yes	No	NA
02	T3	N2	M0	III	SD	T2	N2	M0	IIIB	MPR	No	Yes	Mediastinal lymph nodes
03	T3	N1	M0	III	PR	T3	N1	M0	IIIB	MPR	No	Yes	Supraclavicular lymph node and liver metastasis
04	T3	N0	M0	II	PR	T3	N1	M0	IIIB	PR	No	No	NA
05	T3	N1	M0	III	SD	T3	N1	M0	IIIB	SD	No	No	NA
06	T3	N2	M0	III	PR	T2	N0	M0	I	MPR	Yes	No	NA
07	T3	N0	M0	II	PR	T1a	N0	M0	I	MPR	Yes	No	NA
08	T3	N1	M0	III	PR	T1b	N1	M0	IIIA	PR	No	Yes	Mediastinal lymph nodes
09	T3	N1	M0	III	PR	T2	N0	M0	I	MPR	Yes	No	NA
10	T3	N2	M0	III	PR	T0	N0	M0	I	PCR	Yes	No	NA
11	T2	N1	M0	II	PR	T1a	N0	M0	I	MPR	Yes	No	NA
13	T3	N0	M0	II	PR	T2	N0	M0	I	PR	Yes	Yes	Mediastinal lymph nodes and liver metastasis
14	T3	N0	M0	II	PR	T0	N0	M0	I	PCR	Yes	No	NA
16	T3	N1	M0	III	PR	T2	N0	M0	I	MPR	Yes	Yes	Mediastinal lymph nodes
17	T3	N0	M0	II	CR	T0	N0	M0	I	PCR	Yes	No	NA
18	T3	N1	M0	III	SD	T3	N0	M0	II	SD	Yes	No	NA
20	T3	N1	M0	III	PR	T1a	N0	M0	I	MPR	Yes	No	NA
21	T3	N0	M0	II	PR	T1a	N1	M0	IIIA	MPR	No	No	NA
22	T3	N2	M0	III	PR	T0	N1	M0	IIIA	PCR	No	No	NA
23	T3	N2	M0	III	PR	Tis	N0	M0	I	MPR	Yes	No	NA

Abbreviations: PCR, pathological complete response; MPR, major pathological response; CR, complete response; PR, partial response; SD, stable disease; yp, pathologic stage after neoadjuvant treatment.

Supplemental table S4. Association of patient characteristics with pathological complete response.

	PCR (n=5)	Non-PCR (n=15)	*P* value
Smoking status			**1.000**
Never	1	5	
Former or current	4	10	
Clinical TNM stage			**0.347**
II	3	5	
III	2	10	
Clinical T stage			
2	1	1	**0.447**
3	4	14	
Lymph node metastasis			**0.612**
Yes	3	11	
No	2	4	

Supplemental table S5. Summary of quality of life for all enrolled patients (n=20) before and after chemoimmunotherapy.

Outcome variables	Before neoadjuvant therapy	After neoadjuvant therapy
Quality of Life		
EORTC-QLQ-C30		
Overall quality of life scale[a]	57.92±14.78	81.25±9.82**
Functioning scale[b]		
Physical functioning	92.33±10.39	97.33±7.72*
Role functioning	92.50±14.41	99.17±3.63
Emotional functioning	82.92±13.30	91.67±8.33*
Cognitive functioning	83.33±23.57	98.33±7.26*
Social functioning	81.67±25.22	92.50±12.33
General symptom scales[c]		
Fatigue	18.89±16.14	7.22±11.26**
Nausea and vomiting scale	12.50±14.79	0.83±3.63**
Pain	24.17±17.85	1.67±5.00**

Yang W, *et al. J Immunother Cancer* 2022; 10:e003497. doi: 10.1136/jitc-2021-003497

Dyspnea	10.00±15.27	3.33±10.00
Insomnia	10.00±15.28	8.33±17.87
Appetite loss	11.67±19.07	0.00±0.00*
Constipation	15.00±26.82	3.33±10.00
Diarrhea	11.67±15.90	3.33±10.00
Financial difficulties	38.33±30.32	16.67±26.87*
EORTC-QLQ-OES18		
General functional scales[d]		
Dysphagia	30.55±21.33	6.10±13.37**
General symptom scales[e]		
Difficulty swallowing saliva	11.67±24.21	0.00±0.00*
Choking when swallowing	46.67±30.55	8.33±17.87**
Eating	24.58±18.35	3.75±8.53**
Dry mouth	13.33±19.44	13.33±16.33
Difficulty tasting	11.67±21.79	1.67±7.26
Coughing	1.67±7.26	0.00±0.00
Difficulty talking	0.00±0.00	0.00±0.00
Reflux	18.33±21.67	7.50±13.41
Pain	17.22±15.51	2.78±5.96**

*$P < 0.05$; ** $P < 0.01$.

[a] Higher scores mean better health;

[b,d] Higher scores mean better function;

[c,e] Higher scores mean worse symptoms.

Abbreviations: EORTC-QLQ-C30, European Organization for Research and Treatment of Cancer Quality of life Question-Core-30; OES-18, Esophageal Cancer Module-18.

BMJ Publishing Group Limited (BMJ) disclaims all liability and responsibility arising from any reliance placed on this supplemental material which has been supplied by the author(s)

Supplemental table S6. Association between PD-L1 and/or TMB and downstaging of patients (n=19).

	**Downstaging of TNM stage**		***P* value**
	**Yes (n=12)**	**No (n=7)**	
PD-L1			**1.000**
Positive	8	4	
Negative	4	3	
TMB status			**0.602**
TMB-H	4	1	
TMB-L	8	6	

	**Downstaging of T stage**		***P* value**
	**Yes (n=15)**	**No (n=4)**	
PD-L1			**0.117**
Positive	11	1	
Negative	4	3	
TMB status			**0.530**
TMB-H	5	0	
TMB-L	10	4	

Yang W, *et al. J Immunother Cancer* 2022; 10:e003497. doi: 10.1136/jitc-2021-003497

3. SUPPLEMENTARY FIGURES

Supplemental figure 1. Number of cells per mm^2 in tissue before and after treatment among all patients (n=19) who received surgery based on immunohistochemistry.

(A) The number of CD4$^+$ cells was significantly increased after treatment. (B) The number of CD8$^+$ cells was significantly increased after treatment. (C) The number of CD56$^+$ cells was significantly increased after treatment. (D) The number of PD-1$^+$ cells was significantly increased after treatment. (E) The number of GRB$^+$ cells was significantly increased after treatment. (F) The number of TIA-1$^+$ cells was significantly increased after treatment. (G) No significant change in the number of PD-L1$^+$ cells was observed after chemoimmunotherapy. (H) No significant change in the number of CD163$^+$ cells was observed after chemoimmunotherapy.

**: *P*<0.01; NS: not significant

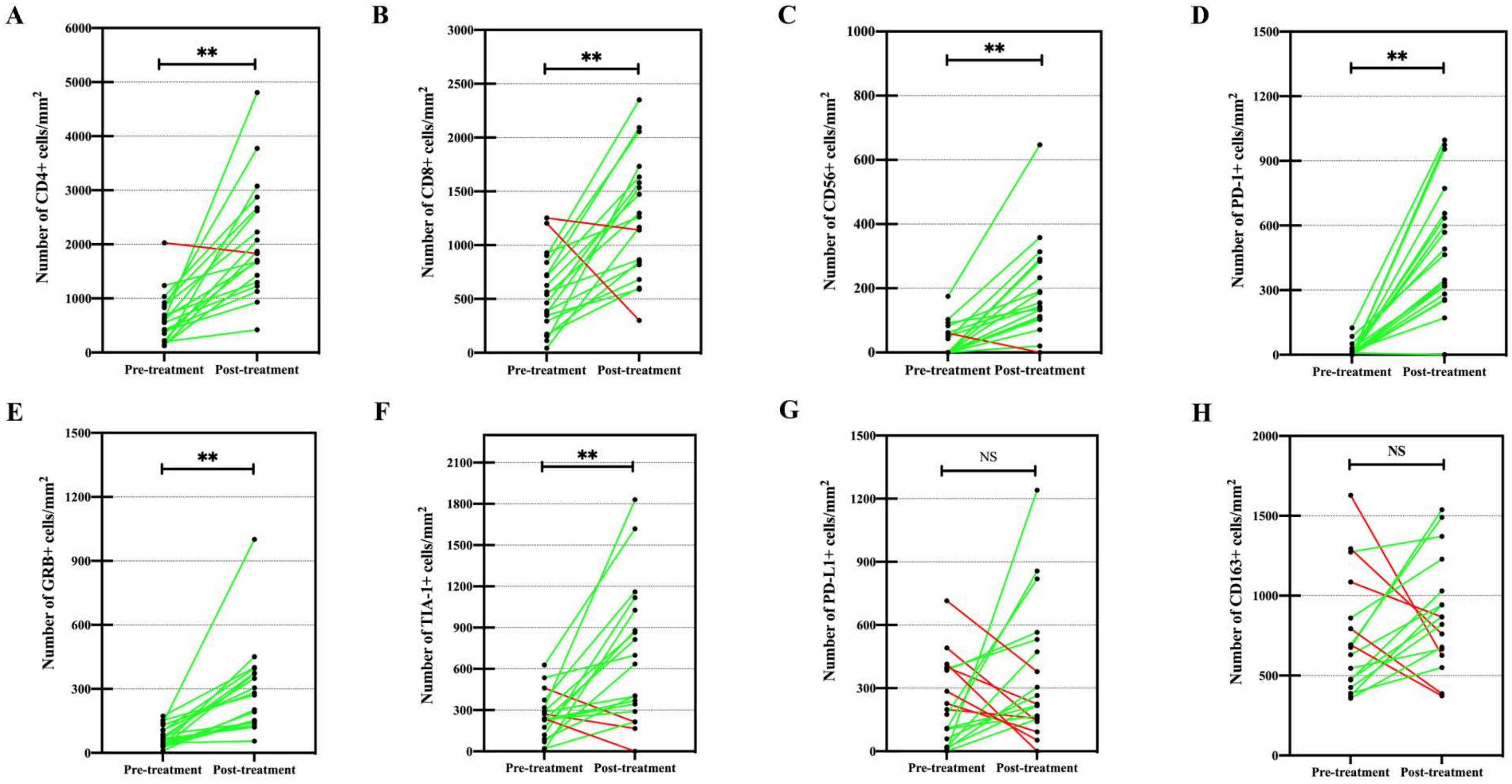

Yang W, *et al. J Immunother Cancer* 2022; 10:e003497. doi: 10.1136/jitc-2021-003497

Supplemental figure 2. Comparison of infiltrating immune cells between the PCR group (n=5) and the non-PCR (n=14) group before and after treatment based on immunohistochemistry.

(A) Comparison of infiltrating CD56^{+} cells between the PCR group (n=5) and the non-PCR group (n=14) before and after treatment.

(B) Comparison of infiltrating PD-1^{+} cells between the PCR group (n=5) and the non-PCR group (n=14) before and after treatment.

(C) Comparison of infiltrating GRB^{+} cells between the PCR group (n=5) and the non-PCR group (n=14) before and after treatment.

(D) Comparison of infiltrating TIA-1^{+} cells between the PCR group (n=5) and the non-PCR group (n=14) before and after treatment.

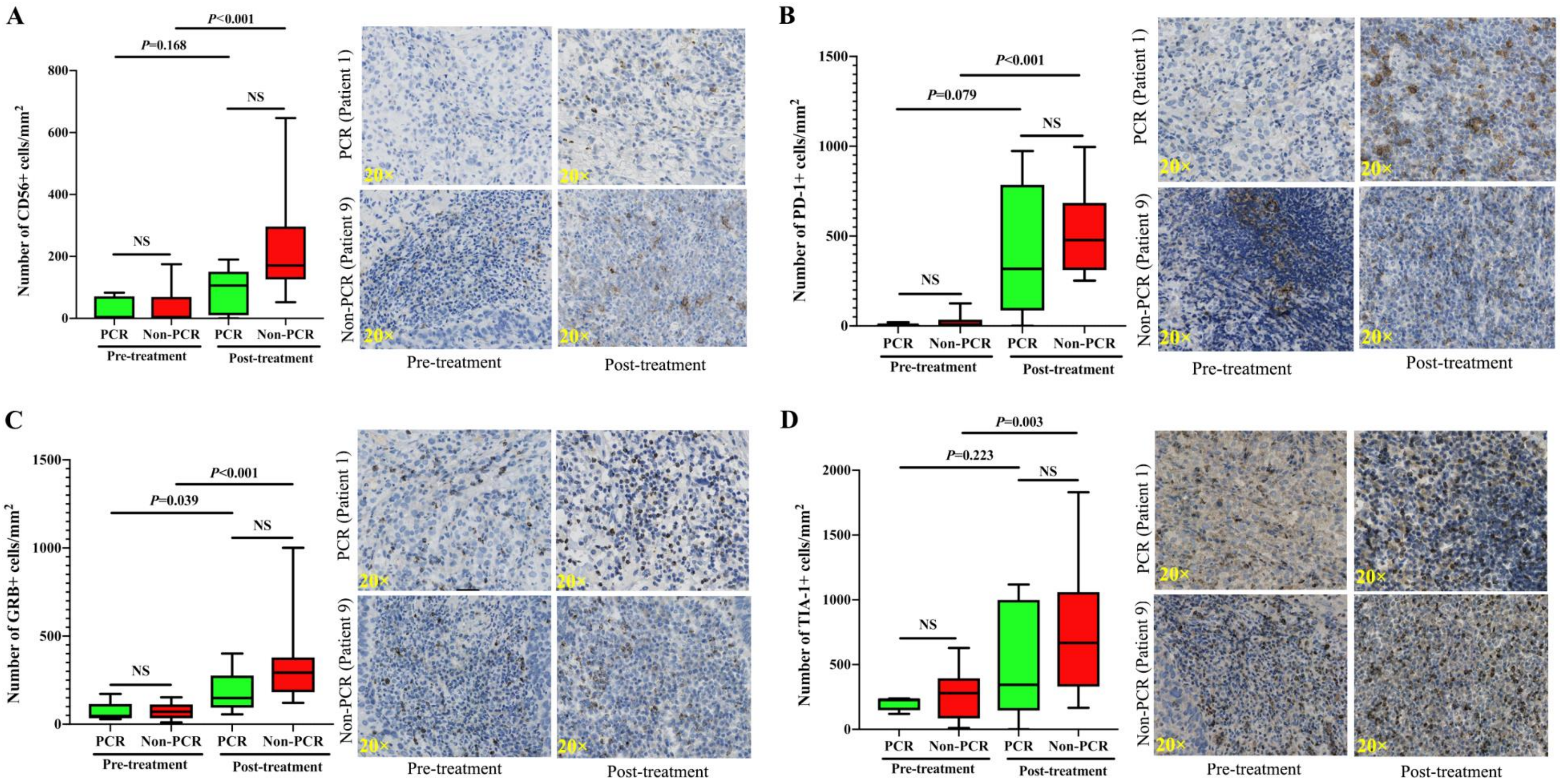

Yang W, *et al. J Immunother Cancer* 2022; 10:e003497. doi: 10.1136/jitc-2021-003497

Supplemental figure 3. Comparison of infiltrating immune cells between the PCR + MPR group (n=14) and the PR+SD (n=5) group before and after treatment based on immunohistochemistry and multiplex immunofluorescence staining.

Comparison of infiltrating CD4$^+$ (A), CD8$^+$ (B), CD56$^+$ (C), PD-1$^+$(D), GRB$^+$ (E), TIA-1$^+$ (F), PD-L1$^+$ (G), CD163$^+$ (H) and CD8$^+$ PD-1$^+$ (I) cells between the PCR + MPR group (n=14) and the PR+SD (n=5) group before and after treatment.

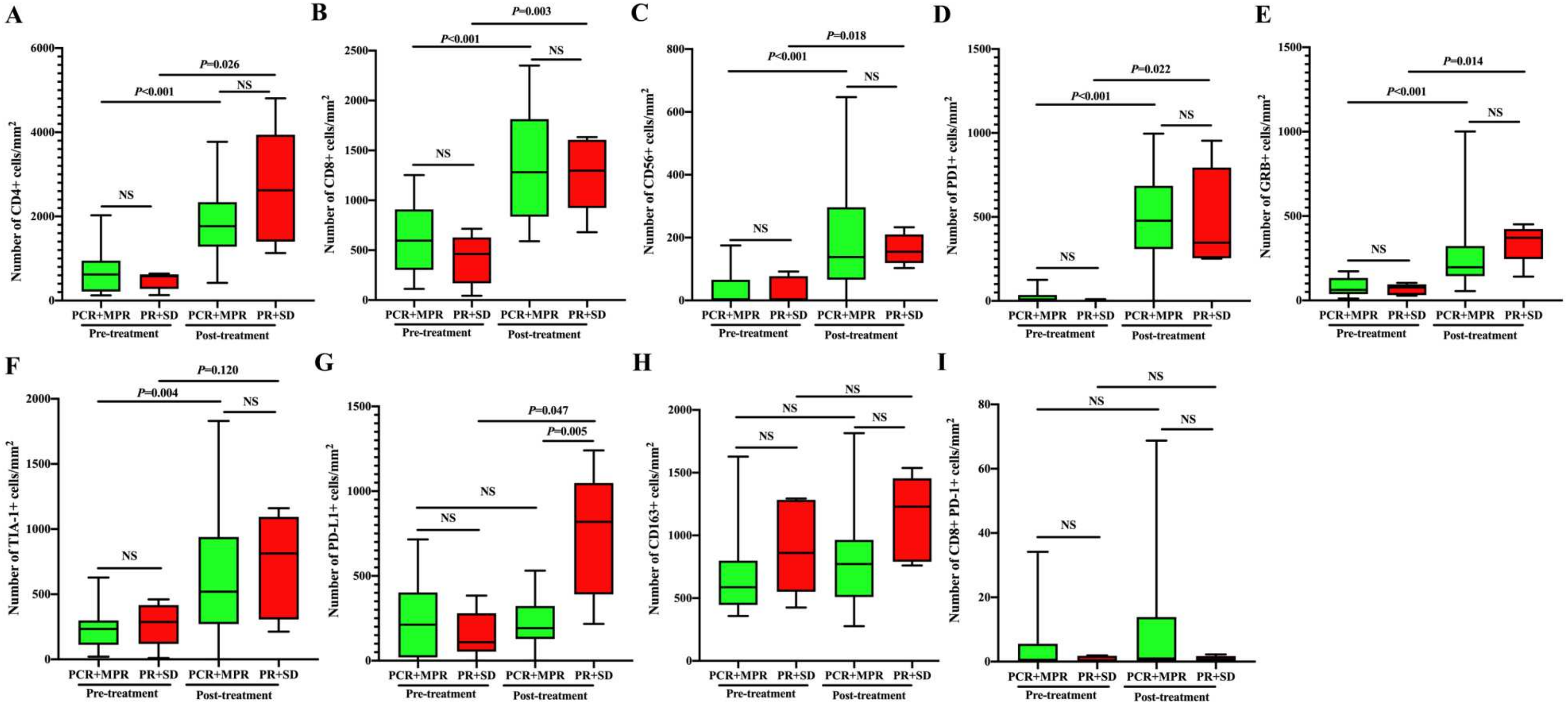

Supplemental figure 4. Comparison of the change in infiltrating immune cells between the PCR group (n=5) and the non-PCR (n=14) group in pre-treatment and post-treatment samples based on immunohistochemistry.

There was no significant difference in the change in infiltrating $CD4^+$ (A), $CD8^+$ (B), $CD56^+$ (C), PD-1^+ (D), GRB^+ (E), TIA-1^+ (F), PD-$L1^+$ (G) and $CD163^+$ (H) between the PCR group (n=5) and the non-PCR group (n=14) before and after treatment. The ratios represented by the Y axis were calculated by the densities of infiltrating immune cells in post-treatment samples divided by the infiltrating immune cells in pre-treatment samples. The ratio >1 means the infiltrating immune cells increase after treatment, while the ratio <1 means the infiltrating immune cells decrease after treatment.

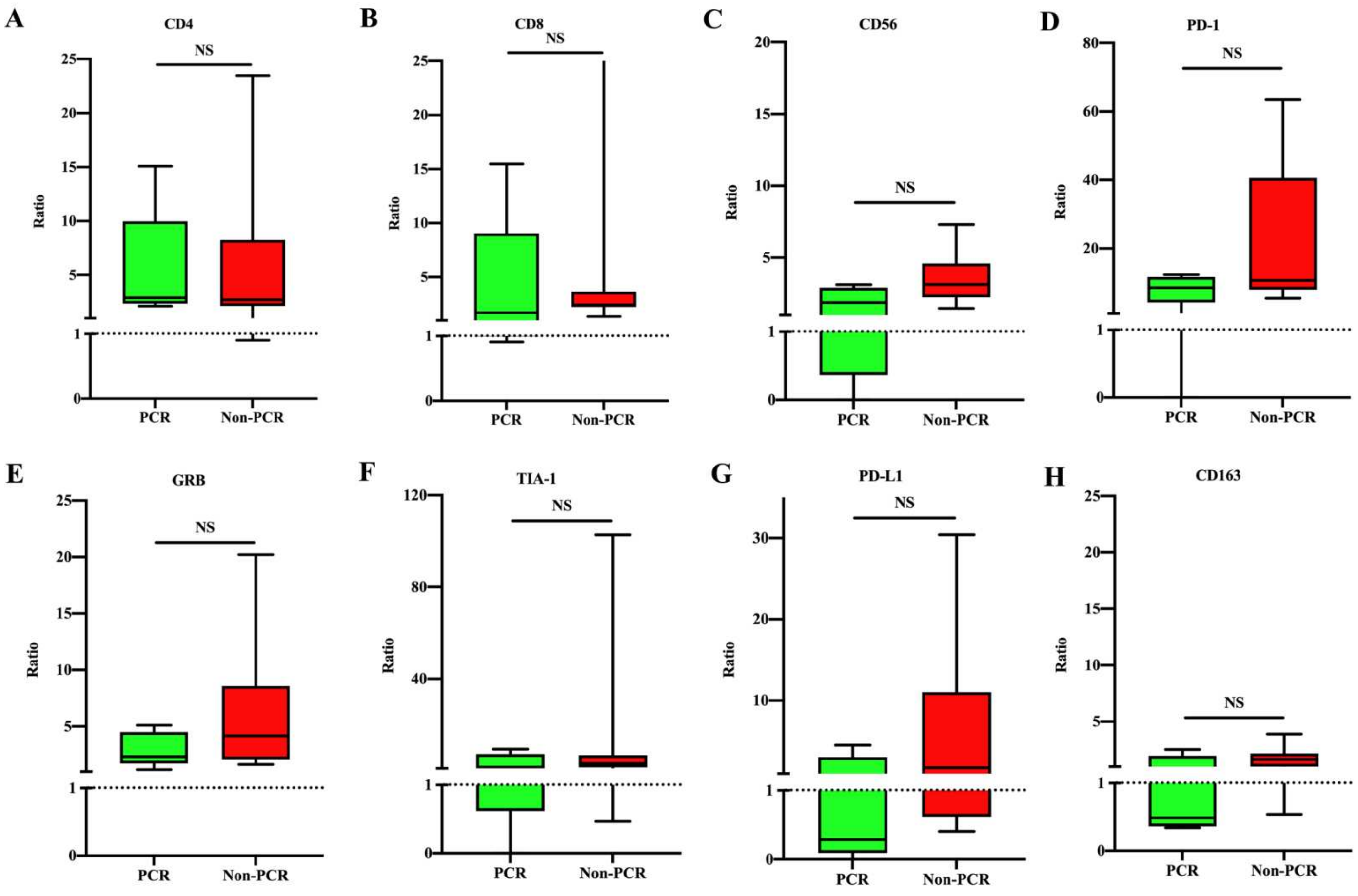

Supplemental figure 5. The correlation analysis between PD-L1 and infiltrating immune cells in pre-treatment and post-treatment samples (n=18) based on multiplex immunofluorescence staining.

(A, B) The number of PD-L1$^+$ cells were positively correlated with CD163$^+$ cells and PD-L1$^+$ CD163$^+$ cells in pre-treatment samples. (C-E) There was no correlation between PD-L1$^+$ cells and infiltrating PD-1$^+$, CD8$^+$ and CD8$^+$ PD-1$^+$ cells in pre-treatment samples. (F) The number of PD-L1$^+$ cells were positively correlated with PD-L1$^+$ CD163$^+$ cells in post-treatment samples. (G-I) There was no correlation between PD-L1$^+$ cells and infiltrating PD-1$^+$, CD8$^+$ and CD8$^+$ PD-1$^+$ cells in post-treatment samples.

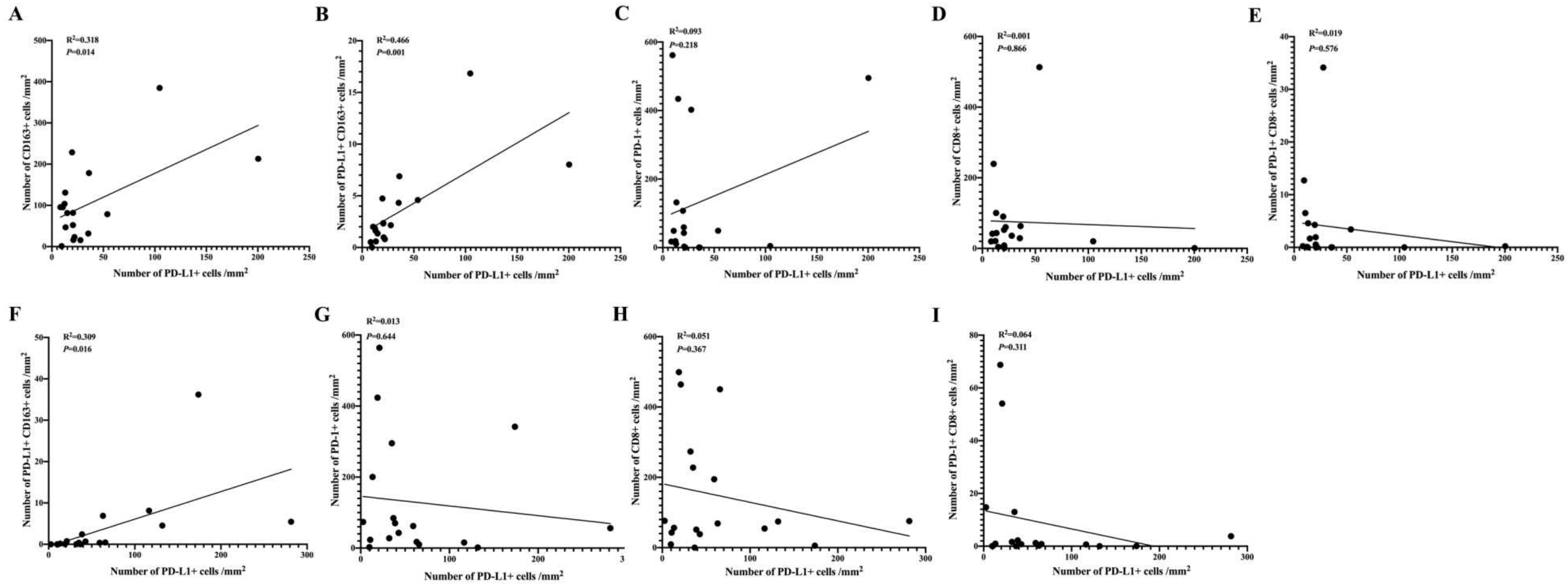

Supplemental figure 6. Comparison of the infiltrating immune cells between the PCR group (n=5) and the non-PCR group (n=13) before and after treatment based on multiplex immunofluorescence staining.

(A) Comparison of the infiltrating immune cells between the PCR group (n=5) and the non-PCR group (n=13) before treatment.

(B) Comparison of the infiltrating immune cells between the PCR group (n=5) and the non-PCR group (n=13) after treatment.

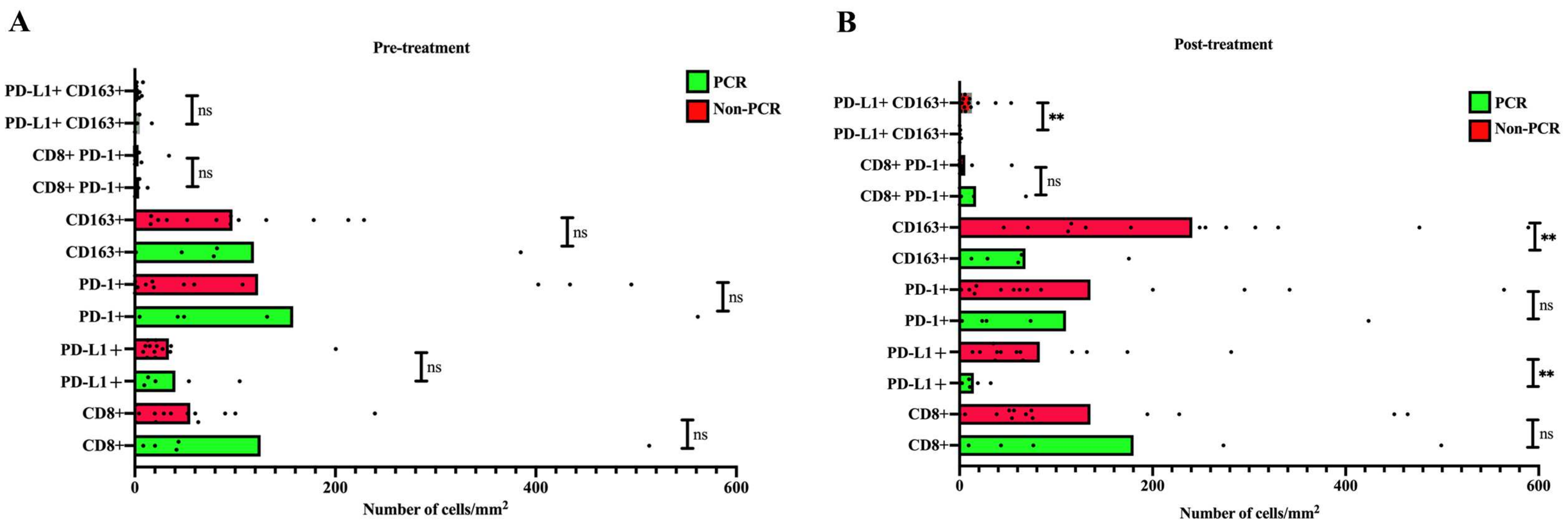

Yang W, *et al. J Immunother Cancer* 2022; 10:e003497. doi: 10.1136/jitc-2021-003497

Supplemental figure 7. Genomic and PD-L1 analyses in the PCR group (n=5) and the non-PCR group (n=14).

(A) The landscape of genomic alterations in the tumors of patients who received surgery before treatment (n=19).

Comparison of TMB (B), PD-L1-CPS (C), and the immune-related pathway (D) between the PCR (n=5) and non-PCR groups (n=14).

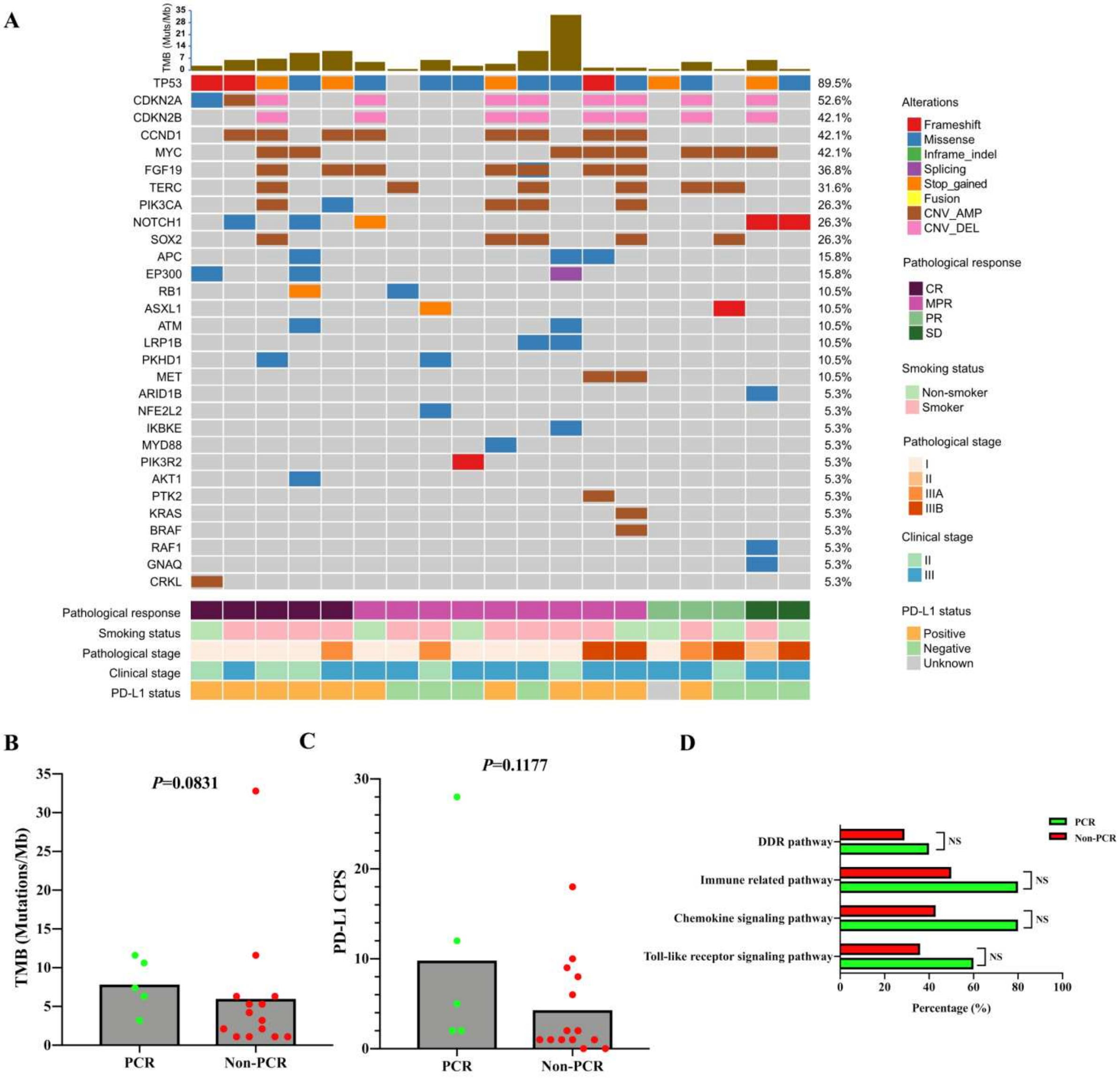

Supplemental figure 8. Disease-free survival (DFS) by different TMB and PD-L1 status (n=19).

(A) DFS by TMB-H (n=5) and TMB-L (n=14).

(B) DFS by PD-L1+ (n=12) and PD-L1- (n=7).

(C) DFS by both TMB-H and PD-L1+ (n=4) and others (n=15).

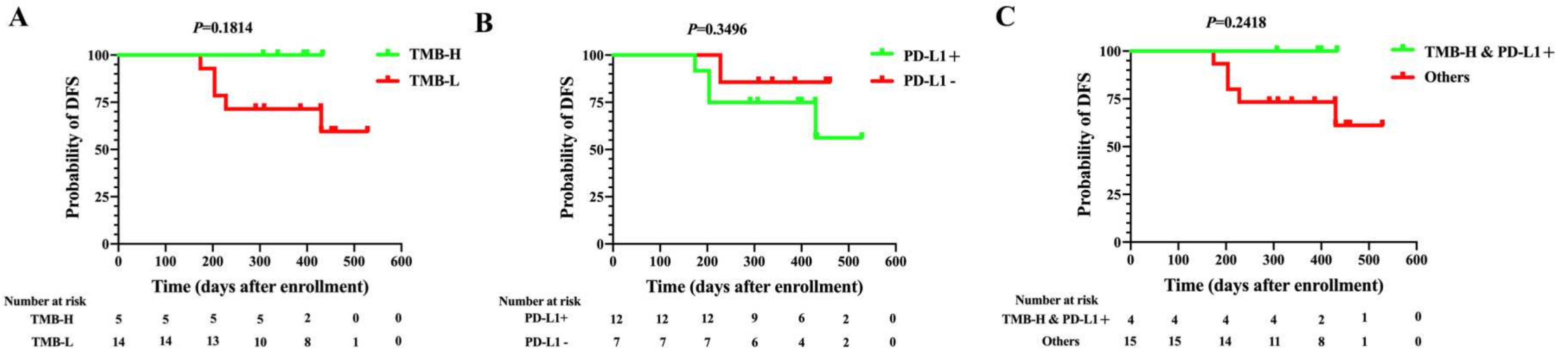

4. SUPPLEMENTARY REFERENCES

1. Aaronson N, Ahmedzai S, Bergman B, et al. The European Organization for Research and Treatment of Cancer QLQ-C30: a quality-of-life instrument for use in international clinical trials in oncology. *Journal of the National Cancer Institute* 1993;85(5):365-76. doi: 10.1093/jnci/85.5.365
2. Blazeby J, Conroy T, Hammerlid E, et al. Clinical and psychometric validation of an EORTC questionnaire module, the EORTC QLQ-OES18, to assess quality of life in patients with oesophageal cancer. *European journal of cancer (Oxford, England : 1990)* 2003;39(10):1384-94. doi: 10.1016/s0959-8049(03)00270-3
3. Lantuejoul S, Sound-Tsao M, Cooper W, et al. PD-L1 Testing for Lung Cancer in 2019: Perspective From the IASLC Pathology Committee. *Journal of thoracic oncology : official publication of the International Association for the Study of Lung Cancer* 2020;15(4):499-519. doi: 10.1016/j.jtho.2019.12.107
4. Kulangara K, Zhang N, Corigliano E, et al. Clinical Utility of the Combined Positive Score for Programmed Death Ligand-1 Expression and the Approval of Pembrolizumab for Treatment of Gastric Cancer. *Archives of pathology & laboratory medicine* 2019;143(3):330-37. doi: 10.5858/arpa.2018-0043-OA
5. Taube J, Klein A, Brahmer J, et al. Association of PD-1, PD-1 ligands, and other features of the tumor immune microenvironment with response to anti-PD-1 therapy. *Clinical cancer research : an official journal of the American Association for Cancer Research* 2014;20(19):5064-74. doi: 10.1158/1078-0432.Ccr-13-3271
6. Nghiem P, Bhatia S, Lipson E, et al. PD-1 Blockade with Pembrolizumab in Advanced Merkel-Cell Carcinoma. *The New England journal of medicine* 2016;374(26):2542-52. doi: 10.1056/NEJMoa1603702
7. Yang Z, Yang N, Ou Q, et al. Investigating Novel Resistance Mechanisms to Third-Generation EGFR Tyrosine Kinase Inhibitor Osimertinib in Non-Small Cell Lung Cancer Patients. *Clin Cancer Res* 2018;24(13):3097-107. doi: 10.1158/1078-0432.Ccr-17-2310 [published Online First: 2018/03/07]
8. Bolger AM, Lohse M, Usadel B. Trimmomatic: a flexible trimmer for Illumina sequence data. *Bioinformatics* 2014;30(15):2114-20. doi: 10.1093/bioinformatics/btu170 [published Online First: 2014/04/04]
9. Koboldt DC, Zhang Q, Larson DE, et al. VarScan 2: somatic mutation and copy number alteration discovery in cancer by exome sequencing. *Genome Res* 2012;22(3):568-76. doi: 10.1101/gr.129684.111 [published Online First: 2012/02/04]
